# Exercise-Induced Hepatic Mitochondrial Reprogramming Across Muscle–Gut–Thyroid Axes in MASLD/MASH

**DOI:** 10.3390/ijms27146112

**Published:** 2026-07-08

**Authors:** Jonas M. McCaffrey, Jamal A. Ibdah

**Affiliations:** 1 School of Medicine, University of Missouri, Columbia, MO 65212, USA; 2 Division of Gastroenterology and Hepatology, University of Missouri, Columbia, MO 65212, USA; 3 Harry S. Truman Memorial Veterans Medical Center, Columbia, MO 65201, USA; 4 Department of Medical Pharmacology and Physiology, University of Missouri, Columbia, MO 65212, USA; 5 Department of Nutrition and Exercise Physiology, University of Missouri, Columbia, MO 65212, USA

**Keywords:** liver, gut, thyroid, mitochondrial dysfunction, exercise, MASLD

## Abstract

Metabolic dysfunction-associated steatotic liver disease (MASLD) and its progressive form, metabolic dysfunction-associated steatohepatitis (MASH), represent a growing global health burden driven by complex interactions among hepatic lipid accumulation, insulin resistance, chronic inflammation, and mitochondrial dysfunction. Exercise remains the cornerstone of lifestyle therapy for MASLD/MASH; however, its therapeutic benefits extend well beyond weight reduction and involve coordinated molecular adaptations across multiple organ systems. In this review, we introduce hepatic mitochondrial reprogramming as a conceptual framework describing the coordinated remodeling of mitochondrial energetics, quality-control pathways, and redox homeostasis that collectively restore metabolic flexibility and hepatocellular resilience. Exercise activates key metabolic regulators, including AMP-activated protein kinase (AMPK), peroxisome proliferator-activated receptor-γ coactivator-1α (PGC-1α), and sirtuin signaling, promoting mitochondrial biogenesis, fatty acid oxidation, oxidative phosphorylation, and mitophagy while suppressing hepatic lipogenesis and oxidative injury. Skeletal muscle-derived myokines, alterations in gut microbial metabolism, and thyroid hormone signaling converge upon hepatic mitochondrial function through complementary endocrine and metabolic pathways. Together, these adaptations reduce hepatic steatosis, lipotoxicity, inflammation, and fibrogenesis while improving insulin sensitivity and metabolic flexibility. Emerging evidence further suggests that exercise-induced mitochondrial remodeling may complement pharmacologic therapies targeting hepatic metabolism, including thyroid hormone receptor-β agonists. Although multi-omics technologies continue to expand our understanding of these adaptive responses, the present review emphasizes the underlying molecular and physiological mechanisms through which exercise remodels hepatic mitochondrial function. We propose that exercise acts as a systems-level mitochondrial remodeling stimulus integrating skeletal muscle-, gut-, and thyroid-derived signals to improve hepatic metabolism and attenuate MASLD/MASH progression. This conceptual framework provides a mechanistic basis for precision exercise prescriptions and future combination therapeutic strategies targeting mitochondrial health.

## 1. Introduction

Metabolic dysfunction-associated steatotic liver disease (MASLD) is characterized by excessive accumulation of intrahepatic triglycerides and may progress to metabolic dysfunction-associated steatohepatitis (MASH) if not addressed [1]. MASH is strongly associated with liver-related morbidity and is histologically defined by steatosis, lobular inflammation, and hepatocyte ballooning, with or without accompanying fibrosis [1]. As of 2023, around 30% of the global population exhibits features consistent with MASLD, with prevalence expected to rise in parallel with increasing rates of obesity, insulin resistance, and physical inactivity [2]. Although pharmacologic therapies like thyroid hormone receptor-β agonists have been recently approved, lifestyle modifications like diet and exercise remain the first-line interventions for MASLD. Clinical exercise interventions reduce intrahepatic lipid content, improve insulin sensitivity, and enhance cardiometabolic health, even in the absence of significant weight loss [3].

At the cellular level, hepatic mitochondria regulate energy production, apoptosis, and lipid oxidation. Disruptions in these processes promote oxidative stress, inflammatory signaling, and lipid accumulation, establishing mitochondrial dysfunction as a defining feature of MASLD. Alterations in mitochondrial function have also been shown to influence MASLD progression from steatosis to fibrosis [4]. Across clinical and preclinical studies, many of the metabolic pathways impaired in MASLD, including fatty acid oxidation, redox balance, and mitochondrial quality control, appear responsive to exercise, suggesting mitochondrial adaptation may contribute to the therapeutic effects of physical activity [5]. Collectively, accumulating evidence suggests that hepatic mitochondria represent an important mechanistic nexus through which exercise coordinates metabolic adaptations across multiple organ systems. Rather than serving solely as cellular energy producers, mitochondria integrate nutrient sensing, oxidative metabolism, redox signaling, inflammatory regulation, and quality-control mechanisms that collectively determine hepatocellular metabolic resilience. Consequently, mitochondrial dysfunction has emerged as a hallmark of MASLD progression and a promising therapeutic target for both lifestyle and pharmacologic interventions.

Exercise affects MASLD through mechanisms that extend beyond caloric expenditure and weight loss. We propose that exercise acts as a systems-level mitochondrial remodeling stimulus that coordinates adaptive signaling across skeletal muscle, liver, gut microbiome, and thyroid hormone pathways. These integrated responses collectively improve substrate handling, reduce lipotoxicity, enhance mitochondrial quality control, and attenuate progression of MASLD/MASH.

Hepatic Mitochondrial Reprogramming: Definition and Conceptual Framework: The concept of hepatic mitochondrial reprogramming forms the central theme of this review. We define hepatic mitochondrial reprogramming as the coordinated adaptive remodeling of mitochondrial structure and function that encompasses substrate utilization, oxidative phosphorylation, mitochondrial biogenesis, mitochondrial dynamics, mitophagy, redox homeostasis, and organelle quality control. Unlike isolated changes in mitochondrial activity or metabolic flexibility, mitochondrial reprogramming represents an integrated systems-level response through which multiple signaling pathways converge to restore mitochondrial efficiency, metabolic resilience, and hepatocellular homeostasis [6,7,8].

Exercise represents one of the most potent physiological stimuli capable of inducing this coordinated remodeling. During repeated bouts of physical activity, metabolic stress activates conserved signaling networks including AMPK, PGC-1α, sirtuins, and thyroid hormone-dependent pathways that collectively regulate mitochondrial energetics, substrate oxidation, mitochondrial turnover, and antioxidant defense. Simultaneously, exercise modifies endocrine and metabolic communication among skeletal muscle, the gastrointestinal tract, adipose tissue, and the liver through myokines, microbial metabolites, bile acid signaling, and hormonal regulation. Rather than functioning as independent pathways, these signals converge upon hepatic mitochondria, where they collectively promote adaptive remodeling of mitochondrial function [6,7,8].

**Figure 1:**
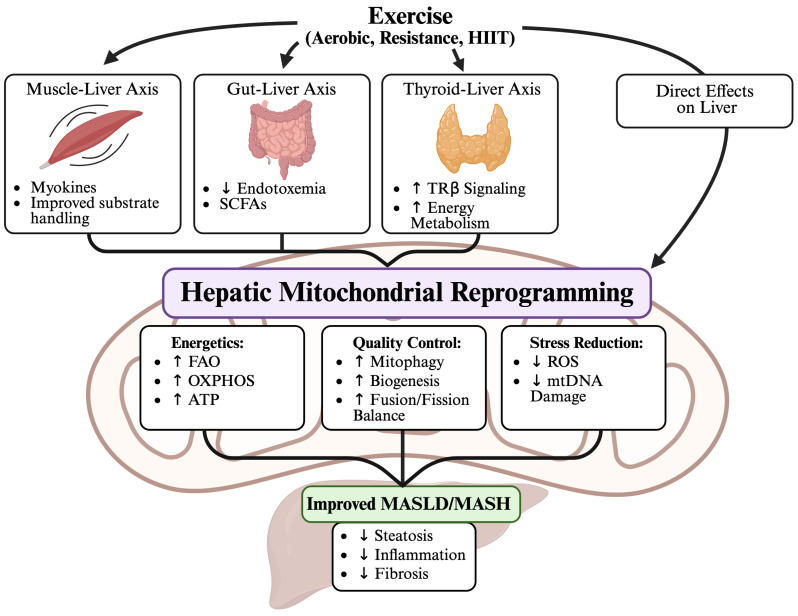
Exercise-Induced Hepatic Mitochondrial Reprogramming: A Systems-Level Framework for MASLD/MASH. ATP: Adenosine Triphosphate; FAO: Fatty Acid Oxidation; HIIT: High-Intensity Interval Training; MASLD: Metabolic Dysfunction-Associated Steatotic Liver Disease; MASH: Metabolic Dysfunction-Associated Steatohepatitis; mtDNA: Mitochondrial DNA; OXPHOS: Oxidative Phosphorylation; ROS: Reactive Oxygen Species; SCFAs: Short-Chain Fatty Acids; TRβ: Thyroid Hormone Receptor Beta. Created in BioRender. Jonas McCaffrey (2026) https://BioRender.com/5otxori (accessed on 4 June 2026).

Accordingly, this review proposes that exercise should be viewed not merely as an intervention that increases energy expenditure or reduces body weight, but as a systems-level regulator of hepatic mitochondrial biology. This conceptual framework provides a unifying mechanism linking skeletal muscle–liver, gut–liver, thyroid–liver, and direct hepatic adaptations into a coherent model explaining how exercise attenuates the progression of MASLD/MASH as illustrated in Figure 1.

In this review, we synthesize current evidence supporting hepatic mitochondrial reprogramming as a unifying mechanistic framework through which exercise influences MASLD/MASH progression. We examine how exercise-induced adaptations originating in skeletal muscle, the gut microbiome, thyroid hormone signaling, and the liver itself converge upon mitochondrial energetics, quality-control pathways, and redox homeostasis to restore metabolic flexibility and improve liver health. We further discuss emerging translational implications of this framework, including precision exercise prescriptions, mitochondrial biomarkers, and combination therapies integrating lifestyle intervention with targeted pharmacologic approaches.

## 2. Pathophysiology of MASLD/MASH: Mitochondrial Dysfunction and Energetic Imbalance

Mitochondrial dysfunction links hepatic lipid accumulation to oxidative stress, inflammation, and impaired cellular energetics in MASLD/MASH. This section reviews normal mitochondrial function and the mechanisms by which metabolic stress disrupts hepatic bioenergetics during disease progression.

### 2.1. Normal Mitochondrial Function and Quality Control

Mitochondria are the primary energy-generating organelles within cells and are central regulators of hepatic substrate metabolism. Acetyl-CoA functions as a key metabolic intermediate that enters the tricarboxylic acid (TCA) cycle to support adenosine triphosphate (ATP) production. Acetyl-CoA is derived from pyruvate during glucose catabolism or from fatty acids through β-oxidation. In hepatocytes, free fatty acids (FFAs) are converted to fatty acyl-CoA in the cytosol and transported into mitochondria through the carnitine shuttle. β-oxidation then generates acetyl-CoA units that enter the TCA cycle and support ATP generation.

ATP production is primarily mediated by the electron transport chain (ETC), located within the inner mitochondrial membrane. The ETC consists of complexes I–V, where NADH and FADH_2_ donate electrons that move through sequential complexes. This electron flow pumps protons from the mitochondrial matrix into the intermembrane space, generating the proton gradient used by complex V to synthesize ATP from adenosine diphosphate and inorganic phosphate. Proton leakage across the inner membrane reduces the mitochondrial membrane potential (ΔΨm) and produces heat rather than ATP. Electron leakage from the ETC, particularly during substrate overload or respiratory chain dysfunction, promotes mitochondrial reactive oxygen species (ROS) generation. Under physiologic conditions, antioxidant systems such as superoxide dismutase and glutathione peroxidase limit oxidative damage; however, persistent ROS excess can injure mitochondrial DNA, proteins, and membranes.

Mitochondrial integrity is maintained through dynamic processes of fusion and fission [9]. Fusion allows partially damaged mitochondria to combine their contents, supporting functional complementation and reducing cellular stress. Mitofusin-1 (Mfn1) and mitofusin-2 (Mfn2) mediate fusion of the outer mitochondrial membrane (OMM) [10], while optic atrophy 1 (Opa1) regulates fusion of the inner mitochondrial membrane (IMM) [11]. In contrast, fission segregates damaged mitochondrial components and facilitates the formation of new mitochondria through division of pre-existing organelles [12]. This process is primarily regulated by dynamin-related protein 1 (Drp1) and mitochondrial fission 1 protein (Fis1) [13,14]. Disruption of the fusion–fission balance has been implicated in multiple diseases, including MASLD, often in response to intracellular or external stressors that promote mitochondrial fragmentation [9].

Mitophagy, a selective form of autophagy, targets damaged mitochondria for degradation, preserving cellular homeostasis and limiting ROS production [15]. In addition to its quality-control function, mitophagy contributes to metabolic regulation by promoting lipid droplet breakdown and facilitating transfer of FFAs to functional mitochondria for energy production. The PTEN-induced kinase 1 (PINK1) Parkin pathway is a key mediator of mitophagy [16]. Loss of mitochondrial membrane potential leads to PINK1 accumulation and recruitment of autophagic machinery to degrade dysfunctional mitochondria.

Mitochondrial biogenesis, the process of generating new mitochondria, requires replication of mitochondrial DNA (mtDNA), transcription of nuclear-encoded genes, and assembly of oxidative phosphorylation (OXPHOS) complexes [17]. Peroxisome proliferator-activated receptor gamma coactivator-1α (PGC-1α) is a central regulator of this process, coordinating expression of nuclear and mitochondrial genes [17]. PGC-1α activates nuclear respiratory factors 1 and 2 (NRF-1 and NRF-2), which induce transcription factor A (TFAM), a direct regulator of the mitochondrial genome [18,19]. PGC-1α also enhances mitochondrial fatty acid oxidation (FAO) through co-activation of peroxisome proliferator-activated receptors α and γ (PPARα and PPARγ), while promoting mtDNA replication and mitochondrial gene expression [20].

PGC-1α activity is closely coordinated with sirtuins (SIRTs), a family of NAD-dependent protein deacetylases that regulate mitochondrial function [21]. SIRT3, one of the most extensively studied members, modulates ATP production by regulating ETC complexes I and II, activates β-oxidation enzymes and acetyl-CoA synthase, and protects against oxidative stress through interactions with superoxide dismutase 2 (SOD2) and isocitrate dehydrogenase 2 (IDH2) [22]. These pathways are regulated by nutrient- and energy-sensing signals, including AMP-activated protein kinase (AMPK), which links cellular energy status to fatty acid oxidation, mitochondrial biogenesis, and autophagy. The AMPK and sirtuin signaling pathways are responsive to energetic stress and thus provide a mechanistic bridge between exercise-induced changes in cellular energy status and mitochondrial remodeling in MASLD.

Beyond stimulating mitochondrial biogenesis, AMPK serves as a master regulator of mitochondrial quality control by coordinating autophagy and mitophagy in response to energetic stress. During exercise, reductions in intracellular ATP and elevations in AMP activate AMPK, which simultaneously suppresses mechanistic target of rapamycin complex-1 (mTORC1) and directly phosphorylates Unc-51-like kinase-1 (ULK1), initiating autophagosome formation and selective removal of dysfunctional mitochondria through mitophagy. This coordinated AMPK–mTOR–ULK1 signaling axis promotes turnover of damaged mitochondria while preserving an efficient mitochondrial network capable of meeting increased energetic demands [23,24].

Efficient mitophagy is increasingly recognized as a critical determinant of hepatic metabolic health. In MASLD/MASH, chronic nutrient excess, insulin resistance, and oxidative stress impair autophagic flux, resulting in accumulation of dysfunctional mitochondria characterized by reduced oxidative phosphorylation, excessive reactive oxygen species (ROS) production, and impaired β-oxidation. Exercise counteracts these abnormalities by restoring mitochondrial quality control, thereby improving respiratory efficiency and limiting oxidative injury. Although most mechanistic evidence has been derived from experimental models, growing preclinical data support activation of the AMPK–mTOR–ULK1 pathway as one of the principal mechanisms through which exercise induces hepatic mitochondrial reprogramming [25].

### 2.2. Pathologic Basis for MASLD/MASH

The pathogenesis of MASLD is currently understood through a “multiple hit” model. This framework incorporates a range of contributing factors including genetic predisposition, insulin resistance, gut microbiota, dietary habits, and physical activity [26]. The extent to which each factor contributes to MASLD differs amongst individuals. Nevertheless, a central feature of MASLD development is excess caloric intake leading to the accumulation of triglycerides (TG) and lipids within adipose tissue [27]. Thus, a main metabolic disturbance in MASLD involves increased lipolysis and elevated levels of circulating non-esterified FFAs. Patients with MASLD exhibit increased plasma concentrations of FFAs, TGs, ceramides, and bile acids, alongside a relative depletion of phospholipids [28]. Hepatic lipid accumulation arises from multiple sources, with approximately 59% of FFAs derived from adipose tissue lipolysis, 26% from de novo lipogenesis (DNL), and 15% from dietary intake [29]. Within the liver, FFAs may be esterified into TGs for storage or exported as very low-density lipoproteins (VLDLs). Pathologic lipid accumulation in the liver is primarily driven by increased visceral adipose tissue lipolysis, upregulated hepatic DNL, and excessive caloric intake [29].

Ceramides represent one of the most biologically active lipotoxic lipid intermediates contributing to progression of MASLD/MASH. In addition to accumulating within hepatocytes during chronic lipid overload, ceramides function as intracellular signaling molecules that impair insulin signaling through inhibition of Akt phosphorylation and activation of protein kinase C isoforms. These changes reduce glucose utilization while simultaneously promoting hepatic gluconeogenesis and lipid accumulation. Ceramides also disrupt mitochondrial fatty acid oxidation through inhibition of carnitine-dependent fatty acid transport, alter mitochondrial membrane integrity, increase mitochondrial permeability, and promote excessive ROS generation [30]. Collectively, these effects create a vicious cycle in which mitochondrial dysfunction further accelerates lipid accumulation, oxidative stress, and hepatocellular injury. Exercise reduces hepatic ceramide content through enhanced fatty acid utilization, improved insulin sensitivity, and restoration of mitochondrial oxidative capacity, thereby interrupting this feed-forward cycle of lipotoxicity and mitochondrial dysfunction [5].

Elevated circulating FFAs in MASLD contribute to the development of peripheral insulin resistance, which contributes to disease progression [31]. Insulin resistance further enhances hepatic de novo lipogenesis (DNL), promotes the release of inflammatory cytokines and adipokines, and disrupts normal regulation of adipose tissue lipolysis. As this process progresses, hepatic lipid accumulation leads to lipotoxicity, resulting in oxidative stress and impaired mitochondrial and cellular function [31]. ROS accumulation induces lipid peroxidation and damages mitochondrial membranes, ultimately triggering hepatocyte injury, apoptosis, and necrosis [32]. In response to this injury, hepatic stellate cells (HSCs) become activated and contribute to fibrosis through differentiation into myofibroblasts during inflammatory signaling.

MASLD is increasingly recognized as a systemic disorder involving dysregulated inter-organ communication [33]. Skeletal muscle dysfunction and reduced physical activity contribute to impaired glucose disposal and increased substrate delivery to the liver, exacerbating hepatic steatosis and insulin resistance. Alterations in gut microbiota are associated with excess caloric intake and increase the translocation of bacterial products into portal circulation. This activates innate immune pathways and promotes inflammation in the liver [34]. Thyroid hormones contribute to hepatic mitochondrial biogenesis, β-oxidation, cholesterol turnover, and bile acid synthesis through thyroid hormone receptor-dependent transcriptional programming. Impaired signaling may therefore reduce hepatic lipid disposal and increase susceptibility to triglyceride accumulation, oxidative stress, and progression of MASLD to MASH.

Collectively, these metabolic, inflammatory, and endocrine disturbances converge on cellular pathways that disrupt mitochondrial function, impair fatty acid oxidation, and promote oxidative stress. These mechanisms position mitochondrial dysfunction as a central link between systemic metabolic dysregulation and hepatic disease progression in MASLD and will be explored further in the following sections.

### 2.3. Mitochondrial Dysfunction in MASLD/MASH Progression

Increased DNL and reduced β-oxidation are key contributors to hepatic lipid accumulation in MASLD. In models of obese rodents, mitochondrial dysfunction has been shown to precede disease onset [35,36]. In patients with MASLD, early increases in hepatic lipid content are associated with a transient rise in mitochondrial activity, which gives way to oxidative stress and impaired mitochondrial function [37]. Both experimental and clinical data demonstrate that defects in mitochondrial FAO promote hepatic steatosis [38,39]. In humans, hepatic mitochondrial FAO is reduced by approximately 40–50% in patients with MASH compared to individuals with normal liver histology [40]. This decline is accompanied by increased mitochondrial ROS production and alterations in mitochondrial quality-control pathways, supporting a strong link between impaired FAO and disease severity.

As mitochondrial FAO becomes compromised, the liver increasingly relies on alternative oxidative pathways, including peroxisomal and cytochrome-mediated fatty acid oxidation. These pathways further increase ROS generation and contribute to the accumulation of toxic byproducts [41]. Excess ROS disrupts the ETC, promotes mitochondrial outer membrane permeabilization (MOMP), alters membrane potential, and damages mitochondrial structure [35]. In MASLD, respiratory chain activity is significantly reduced across ETC complexes I–V [42]. ROS also induces oxidative damage to mtDNA, increasing mutational burden in ETC-related genes, which worsens with disease severity [43]. Ultrastructural mitochondrial abnormalities, including cristae disruption, matrix rarefaction, and organelle swelling, have been observed in both animal models and human MASLD, alongside elevated ROS and mtDNA damage [36,44].

Together, these findings suggest that mitochondrial dysfunction is not simply a downstream consequence of lipid accumulation. Rather, impaired FAO, ETC dysfunction, ROS excess, and mtDNA injury create a bioenergetic environment that actively promotes hepatic stress and disease progression.

Excess mitochondrial ROS also converts metabolic stress into inflammatory and cell-death signaling. Increased oxidative stress induces expression of pro-inflammatory cytokines such as TNF-α and Fas ligand through signaling pathways including NF-κB and JNK [45]. ROS also activates the NLRP3 inflammasome, promoting production of cytokines including IL-1β, IL-6, and TNF-α [46]. These processes enhance hepatocyte apoptosis and necrosis, in part through cytochrome c release and mitochondrial permeability transition pore (mPTP) formation, which facilitates mtDNA release and amplifies inflammatory signaling [47,48]. Collectively, these mechanisms position mitochondrial dysfunction as a central driver of inflammatory amplification, hepatocellular injury, and progression from steatosis toward MASH and fibrosis.

Beyond activation of the NLRP3 inflammasome, damaged mitochondria initiate innate immune responses through release of mitochondrial damage-associated molecular patterns (mtDAMPs), including mitochondrial DNA (mtDNA), cardiolipin, ATP, and mitochondrial peptides [49,50]. Oxidative injury and mitochondrial membrane permeabilization facilitate cytosolic release of mtDNA, which activates cyclic GMP–AMP synthase (cGAS) and stimulator of interferon genes (STING), leading to induction of type I interferon signaling and amplification of chronic hepatic inflammation [51]. In parallel, mitochondrial antiviral signaling protein (MAVS), located on the outer mitochondrial membrane, serves as an important signaling platform linking mitochondrial stress to activation of NF-κB and interferon regulatory pathways.

These innate immune pathways further integrate mitochondrial dysfunction with hepatocellular injury by sustaining inflammatory signaling, immune cell recruitment, and fibrogenesis during progression from steatosis to MASH. Although much of the current evidence derives from experimental studies, increasing recognition of mtDNA-mediated inflammatory signaling highlights mitochondria as both metabolic organelles and central regulators of hepatic immune homeostasis [49,50,51]. Suppression of excessive mtDAMP release may therefore represent an additional mechanism through which exercise attenuates chronic hepatic inflammation while preserving adaptive mitochondrial signaling.

Disruption of mitochondrial quality-control mechanisms further exacerbates dysfunction in MASLD. AMPK signaling, which regulates mitochondrial homeostasis through downstream effectors PGC-1α and SIRT3, is reduced in MASLD [52]. Decreased expression of PGC-1α is associated with impaired mitochondrial biogenesis and reduced hepatic respiration, while downregulation of sirtuins leads to hyperacetylation of mitochondrial proteins and impaired function [53]. Conversely, increased SIRT3 expression has been shown to enhance mitochondrial respiration, improve redox balance, and reduce hepatic lipid accumulation and oxidative stress [54].

Alterations in mitochondrial dynamics and mitophagy also contribute to disease progression. Dysregulation of fusion and fission proteins has been observed in both human and animal models of MASLD, including reduced expression of Mfn2, which is associated with increased inflammation, triglyceride accumulation, fibrosis, and hepatocarcinogenesis [55]. Impaired mitophagy further promotes the accumulation of damaged mitochondria, as demonstrated in models with reduced PINK1 and Parkin expression, leading to increased apoptosis and mPTP activation [56]. Similarly, defects in mitophagy, such as those observed in Bnip3-deficient models, are associated with decreased mitochondrial membrane potential, abnormal morphology, reduced respiration, and increased ROS and inflammation [57].

In MASLD, impaired FAO, ROS excess, inflammatory activation, and defective mitochondrial turnover reinforce one another, creating a feed-forward cycle of hepatocellular stress. This cycle links lipid overload to impaired respiration, oxidative injury, inflammasome activation, cell death, and failed organelle renewal. Importantly, this vulnerability also provides a therapeutic entry point. Because exercise targets several components of this cycle, including substrate oxidation, redox balance, mitochondrial dynamics, and mitophagy, mitochondrial dysfunction provides a mechanistic rationale for viewing exercise as a mitochondrial remodeling intervention in MASLD/MASH.

Recent evidence suggests that ferroptosis, an iron-dependent form of regulated cell death driven by uncontrolled lipid peroxidation, contributes to progression of MASLD/MASH [58]. Unlike apoptosis or necrosis, ferroptosis is characterized by depletion of glutathione, impaired glutathione peroxidase-4 (GPX4) activity, excessive phospholipid peroxidation, and profound mitochondrial structural abnormalities including condensed mitochondria with disrupted cristae. Oxidative stress generated by dysfunctional mitochondria amplifies lipid peroxidation and sensitizes hepatocytes to ferroptotic cell death, thereby linking mitochondrial dysfunction directly to inflammation and fibrogenesis [59]. Although direct evidence demonstrating exercise-mediated regulation of ferroptosis in human MASLD remains limited, preclinical studies suggest that improvements in mitochondrial antioxidant capacity, redox homeostasis, and lipid metabolism may reduce susceptibility to ferroptotic injury [58,60]. Future investigation of the interaction between exercise-induced mitochondrial reprogramming and ferroptosis may provide additional mechanistic insight into how exercise protects against progression from simple steatosis to advanced steatohepatitis and fibrosis.

## 3. Exercise and Hepatic Energetics

Exercise improves hepatic metabolism through effects that extend beyond weight loss. Adaptations include enhanced fatty acid oxidation, improved redox balance, mitochondrial remodeling, and activation of quality-control pathways that protect against metabolic stress.

### 3.1. Clinical Benefits of Exercise in MASLD

Diet and exercise remain first-line recommendations for MASLD management, but the benefits of exercise extend beyond energy expenditure and weight reduction to include improved substrate partitioning, insulin sensitivity, and hepatic metabolic stress. Current guidelines recommend patients with MASLD engage in more than 150 min of moderate-intensity exercise or more than 75 min of vigorous-intensity exercise per week [61]. To optimize adherence and long-term effectiveness, exercise prescriptions should be individualized based on patient-specific factors such as social support, functional ability, and daily living [62]. Mechanistically, these recommendations are relevant because exercise redistributes excess substrates away from hepatic storage and toward skeletal muscle oxidation, thereby reducing the lipid and glucose burden that contributes to hepatic steatosis and mitochondrial stress [63].

Sedentary behavior is strongly associated with MASLD prevalence, with increased sedentary time correlating positively with disease prevalence in a cohort of Chinese males [64]. Individuals with MASLD also spend more time sedentary than healthy controls and exhibit reduced daily step counts, total daily energy expenditure, and metabolic equivalents (METs), particularly in association with higher BMI and body weight [65,66]. These findings suggest that physical inactivity may worsen MASLD not only by lowering energy expenditure, but also by limiting peripheral substrate utilization and increasing reliance on hepatic lipid handling.

Clinical exercise interventions support this concept. In a randomized clinical trial of patients with MASLD, both moderate-intensity aerobic exercise and resistance exercise improved intrahepatic fat and insulin resistance when added to dietary intervention [67]. Resistance exercise has also been shown to reduce liver fat independent of weight loss in patients with MASLD [68]. Because skeletal muscle is a major site of insulin-mediated glucose disposal, improved peripheral substrate handling may reduce hepatic exposure to glucose and lipid substrates that contribute to de novo lipogenesis and intrahepatic triglyceride accumulation [69].

Clinical and experimental studies consistently demonstrate that regular physical activity improves liver histology and metabolic parameters in MASLD. Aerobic and resistance exercise interventions, both with [70,71], and without weight loss [72,73], are associated with improved cardiorespiratory fitness and significant reductions in intrahepatic triglyceride content compared to standard care. Exercise volume also plays a critical role; achieving greater than 750 metabolic equivalents per week has been associated with a 3.5-fold higher likelihood of clinically meaningful liver fat reduction [70]. While adherence remains the primary determinant of success, exercise intensity may further modulate outcomes. Both moderate and high-intensity exercise have been shown to improve hepatic steatosis [74,75], with higher-intensity protocols often associated with greater reductions in disease prevalence. In clinical exercise studies, high-intensity interval training (HIIT) has emerged as a feasible and effective modality for improving liver health in MASLD, with reported reductions in intrahepatic triglycerides and visceral adiposity that may reflect increased energy expenditure and improved substrate oxidation [76]. Notably, even lower-dose and lower-intensity exercise interventions have demonstrated reductions in hepatic and visceral fat independent of weight loss [77].

Taken together, these clinical findings support exercise as more than a behavioral strategy for reducing hepatic fat. Improvements in liver fat, insulin sensitivity, visceral adiposity, and cardiorespiratory fitness suggest that exercise alters the systemic metabolic environment in which MASLD develops. By increasing skeletal muscle substrate uptake and oxidation, improving insulin responsiveness, and reducing hepatic exposure to excess lipid and glucose flux, exercise may decrease the metabolic pressure placed on hepatic mitochondria. This provides an important bridge to the mitochondrial adaptations discussed below, including changes in hepatic fatty acid oxidation, redox balance, respiratory efficiency, and mitochondrial quality control.

### 3.2. Effects of Exercise on Hepatic Mitochondrial Function

Reduced hepatic mitochondrial oxidative capacity increases susceptibility to hepatic steatosis and liver injury [36]. Exercise training has therefore been investigated as a modulator of hepatic mitochondrial function, although its effects vary depending on disease context, exercise duration, and the mitochondrial outcome measured. Across available studies, exercise does not uniformly increase hepatic mitochondrial mass or resting oxidative capacity, but instead appears to improve mitochondrial stress tolerance, respiratory efficiency, redox balance, and quality control.

In rodent models, short-term endurance training alone may not substantially increase baseline hepatic mitochondrial function or OXPHOS activity; however, it can preserve mitochondrial function under conditions of hepatic stress, including toxic injury [78]. These findings suggest that exercise may improve hepatic mitochondrial resilience even when resting measures of oxidative capacity are modest. Other preclinical studies demonstrate more direct effects on hepatic respiration. In an endurance-training model, 4 weeks of exercise increased hepatic OXPHOS activity, particularly through complex I-linked substrates, without clear increases in mitochondrial biogenesis markers [79]. Exercise has also been associated with favorable adaptations in ETC function, including modulation of complexes I, IV, and V, alongside reduced oxidative stress without increased ROS production [80]. Similarly, swimming-based exercise reduced hepatic mitochondrial oxidative stress through upregulation of antioxidant enzymes [81]. In rodent models, exercise alone has also enhanced mitochondrial function more effectively than pharmacologic intervention with metformin or combined therapy [82]. Together, these findings suggest that exercise may improve hepatic mitochondrial function less by simply expanding mitochondrial content and more by enhancing ETC efficiency, antioxidant defense, and resistance to metabolic stress.

Intrinsic aerobic capacity provides additional, indirect support for the relationship between mitochondrial fitness and liver metabolic resilience. Animal models with high intrinsic aerobic capacity exhibit greater hepatic mitochondrial content and oxidative capacity compared with low-capacity counterparts [83]. Even under metabolic stress, such as experimental ethanol exposure, these animals demonstrate relative resistance to severe liver injury despite increased steatosis [83]. Although intrinsic-capacity models should not be interpreted as direct evidence of exercise-induced protection in MASLD, they support the broader concept that greater mitochondrial capacity and oxidative reserve may reduce susceptibility to metabolic liver injury.

Exercise may also influence mitochondrial efficiency through remodeling of ETC organization. Respiratory complexes can assemble into supercomplexes, which stabilize electron transport and may reduce electron leak during OXPHOS. Exercise training has been implicated in promoting functional supercomplex assembly in humans, where training increased supercomplex formation alongside enhanced mitochondrial respiration [84]. Although much of the available exercise evidence for supercomplex remodeling comes from skeletal muscle, these findings provide a mechanistic framework for how training may improve oxidative efficiency in metabolically stressed tissues, including the liver. Conceptually, this suggests that exercise-induced mitochondrial adaptation may involve qualitative improvements in electron transport organization rather than only quantitative increases in mitochondrial abundance.

Emerging evidence suggests that exercise may improve mitochondrial respiratory efficiency not only by increasing mitochondrial content but also through structural remodeling of the electron transport chain (ETC). Assembly of individual respiratory complexes into higher-order supercomplexes, or respirasomes, is thought to facilitate electron transfer, minimize electron leak, and reduce excessive ROS production while improving ATP generation [85]. Experimental studies in skeletal muscle consistently demonstrate that endurance exercise enhances respiratory supercomplex assembly, contributing to improved oxidative efficiency and metabolic flexibility [84].

However, considerably less information is available regarding respiratory supercomplex remodeling in the liver. Direct evidence supporting exercise-induced reorganization of hepatic ETC supercomplexes remains limited, particularly in patients with MASLD/MASH. Most mechanistic insights derive from animal models or extrapolation from skeletal muscle physiology. Therefore, while respiratory supercomplex remodeling represents an attractive mechanism by which exercise could improve hepatic mitochondrial efficiency, its contribution to human liver disease requires further investigation. Future studies utilizing high-resolution respirometry, cryo-electron microscopy, and human liver tissue will be important for establishing the physiological significance of respiratory supercomplex dynamics in MASLD/MASH.

Exercise may also alter the size and transcriptional capacity of the hepatic mitochondrial network. PGC-1α is a key transcriptional coactivator in this response, linking contractile and endocrine exercise signals to nuclear and mitochondrial programs that support oxidative metabolism [86]. Skeletal muscle contraction during exercise leads to the release of myokines, which act on the liver and converge on pathways that activate PGC-1α. Increases in hepatic PGC-1α expression have been observed even following a single bout of exercise [87,88]. In addition to its role in biogenesis, PGC-1α has been implicated in the regulation of hepatic autophagy and mitophagy, although the underlying mechanisms remain incompletely defined [87]. These findings position PGC-1α as a signaling node through which acute exercise may initiate hepatic mitochondrial transcriptional and quality-control responses.

With repeated stimulation, acute signaling events may translate into more sustained remodeling of the hepatic mitochondrial network. Chronic exercise induces broader adaptations, including changes in mitochondrial content, dynamics, and quality control. In rodent models, both voluntary physical activity and endurance training increase the expression of proteins involved in mitochondrial biogenesis and dynamics, while simultaneously enhancing hepatic autophagy signaling [89]. In rodent models of MASLD induced by high-fat diet (HFD), these exercise interventions mitigate disease progression, improve mitochondrial bioenergetics, and enhance respiratory control ratios. Endurance training has been shown to reverse mitochondrial abnormalities associated with liver disease [90], while voluntary wheel running improves mitochondrial dysfunction induced by HFD [91]. Exercise has also been shown to improve markers of hepatic mitophagy and mitochondrial quality control in HFD-induced rodent models, particularly when combined with diet-induced weight loss [92]. Collectively, these data suggest that chronic exercise reinforces mitochondrial function by coupling improved respiration with enhanced organelle turnover and network remodeling.

Exercise-mediated improvements in mitochondrial structure may further reinforce these functional benefits. Modulation of membrane lipid composition, including improvements in the phosphatidylcholine-to-phosphatidylethanolamine ratio, has been observed with exercise despite high-fat feeding [90]. Because IMM lipid composition influences cristae organization, ETC stability, and OXPHOS efficiency, exercise-induced preservation of membrane structure may represent an additional mechanism by which training supports hepatic bioenergetics during lipid overload. Endurance training in MASH models reduces susceptibility to mitochondrial permeability transition pore (mPTP) opening and favorably regulates proteins involved in mitochondrial fusion and mitophagy, including Bcl-2, Mfn1, Mfn2, and PINK1 [56]. These adaptations promote mitochondrial turnover and network integrity. Consistent with this, moderate physical activity enhances basal hepatic autophagy supporting a protective role of exercise through reductions in lipid accumulation, oxidative stress, and mitochondrial dysfunction [93].

Importantly, the intensity of exercise influences the magnitude and nature of these adaptations. Lower-intensity activity preferentially utilizes lipids as a fuel source, whereas higher-intensity exercise relies more heavily on glucose metabolism [94]. In training models, moderate- and high-intensity exercise increase markers of hepatic autophagy and reduce serum triglyceride levels [95], suggesting a role for autophagy in regulating hepatic lipid metabolism. Acute exercise also induces transient increases in hepatic PGC-1α expression, which returns to baseline within hours [96]. Repeated activation of these pathways likely contributes to sustained improvements in mitochondrial biogenesis and turnover with chronic exercise [97,98]. Thus, exercise dose and intensity may shape whether hepatic adaptations are primarily substrate-driven, transcriptional, or quality-control oriented.

Overall, exercise appears to target several mitochondrial abnormalities implicated in MASLD, including impaired respiration, oxidative stress, defective mitochondrial turnover, and altered membrane structure. The available evidence suggests that these benefits are context dependent: acute exercise may transiently activate transcriptional and quality-control pathways, whereas chronic training produces broader changes in mitochondrial bioenergetics, dynamics, and resilience to metabolic stress. Although many mechanistic data come from preclinical models, these findings support viewing exercise as a mitochondrial reprogramming stimulus rather than solely a caloric expenditure intervention in MASLD.

Exercise-induced improvements in metabolic flexibility extend beyond enhanced substrate utilization to encompass coordinated remodeling of mitochondrial quality-control pathways [22]. During chronic metabolic stress, hepatocytes progressively lose the capacity to appropriately transition between carbohydrate and lipid oxidation, resulting in incomplete fatty acid oxidation, accumulation of lipotoxic intermediates, and increased oxidative stress. Repeated exercise restores this adaptive capacity by simultaneously improving mitochondrial biogenesis, mitophagy, mitochondrial dynamics, and respiratory efficiency [99]. Collectively, these adaptations enable hepatocytes to more effectively respond to fluctuating nutrient availability while maintaining ATP production and limiting ROS generation. Thus, restoration of metabolic flexibility should be viewed not merely as a metabolic consequence of exercise but as an integral component of hepatic mitochondrial reprogramming.

## 4. Skeletal Muscle–Liver Crosstalk in MASLD and Exercise

Skeletal muscle regulates hepatic metabolism through glucose disposal, fatty acid utilization, substrate exchange, and exercise-induced myokine signaling. In MASLD, impaired muscle metabolism may increase hepatic lipid and glucose delivery, contributing to steatosis, insulin resistance, and mitochondrial stress. Exercise may counter these effects by improving skeletal muscle oxidative capacity and restoring endocrine signals that support hepatic lipid handling and mitochondrial adaptation.

### 4.1. Skeletal Muscle as a Metabolic and Endocrine Organ

Skeletal muscle comprises approximately 40% of total body mass and represents the largest protein reservoir in the body, accounting for 50–75% of total protein content [100]. Beyond its structural role, skeletal muscle is a major regulator of systemic metabolism, accounting for most insulin-mediated glucose disposal and contributing substantially to whole-body energy expenditure. As the principal site of contraction-induced substrate utilization, changes in muscle insulin sensitivity and oxidative capacity can directly alter the amount of glucose and lipid delivered to the liver [101].

The muscle–liver axis operates through coordinated substrate exchange and endocrine signaling [102]. During exercise, skeletal muscle increases glucose uptake and fatty acid utilization while releasing metabolites such as lactate, succinate, and malate into circulation. These metabolites are taken up by the hepato-splanchnic circulation, where they function not only as substrates but also as signaling intermediates that regulate hepatic transcriptional programs and energy metabolism [103]. Skeletal muscle also functions as an endocrine organ through secretion of myokines, which mediate inter-organ communication and contribute to systemic metabolic regulation [104]. Through these substrate and endocrine signals, contracting muscle influences hepatic glucose production, lipid oxidation, and stress-response pathways.

In MASLD, impaired muscle metabolism may increase hepatic substrate burden, whereas exercise-induced substrate utilization and myokine release may shift hepatic metabolism toward improved lipid handling and mitochondrial adaptation.

### 4.2. Dysfunction of the Muscle–Liver Axis in MASLD

Disruption of skeletal muscle metabolism due to physical inactivity, aging, or nutrient excess impairs glucose disposal and alters substrate flux, leading to increased delivery of lipids and glucose to the liver. This contributes to hepatic steatosis, insulin resistance, and metabolic dysfunction, highlighting the importance of coordinated muscle–liver communication in maintaining metabolic homeostasis [105].

Sarcopenia is a progressive disorder characterized by loss of skeletal muscle mass, strength, and function. It is associated with MASLD across multiple clinical studies and meta-analyses [106,107]. Clinically, sarcopenia is present in approximately 20–40% of MASLD patients and is associated with increased risk of steatosis, fibrosis progression, and mortality, highlighting its role as a determinant of disease severity [108,109]. Both conditions share overlapping pathophysiological mechanisms, including insulin resistance, chronic inflammation, and physical inactivity [110]. The relationship between sarcopenia and MASLD has been proposed as bidirectional, with each condition exacerbating the other through shared metabolic and inflammatory pathways [111]. Sarcopenia may amplify MASLD by reducing skeletal muscle metabolic capacity, limiting peripheral glucose disposal and fatty acid oxidation, and increasing substrate delivery to the liver. Conversely, MASLD-related inflammation and ectopic lipid accumulation may further impair muscle metabolism, reinforcing a bidirectional cycle of insulin resistance, lipotoxicity, and mitochondrial stress.

Impaired substrate handling driven by insulin resistance links skeletal muscle dysfunction to hepatic disease. In insulin-resistant muscle, defects in insulin signaling reduce GLUT4-mediated glucose uptake and glycogen synthesis, leading to hyperglycemia and compensatory hyperinsulinemia [112]. Excess substrates are consequently redirected to the liver, where they promote de novo lipogenesis and triglyceride accumulation [113]. Concurrently, intramuscular lipid accumulation generates lipotoxic intermediates that further impair insulin signaling and increase circulating free fatty acid flux to the liver [114]. This maladaptive shift in substrate partitioning may contribute to progression from steatosis toward steatohepatitis by increasing hepatic lipid accumulation and lipotoxic stress.

Myosteatosis is an underrecognized driver of this process. Independent of muscle mass, intramyocellular lipid accumulation is associated with early MASH and fibrosis progression, even after adjustment for adiposity and metabolic factors [115,116]. Mechanistically, myosteatosis amplifies systemic lipotoxicity by impairing muscle oxidative capacity and insulin signaling, thereby increasing circulating FFAs and promoting hepatic inflammation and fibrosis [116]. These findings suggest that impaired muscle quality, rather than reduced muscle mass alone, is a key contributor to MASLD progression.

At the level of skeletal muscle, sarcopenia is driven by an imbalance in protein turnover characterized by reduced protein synthesis and increased proteolysis. Dysregulation of anabolic signaling pathways limits muscle protein synthesis, while activation of catabolic pathways promotes degradation of contractile proteins [117,118]. Impaired autophagy further contributes to accumulation of dysfunctional mitochondria, oxidative stress, and cellular damage [118]. These alterations exacerbate metabolic dysfunction through reduced glucose disposal and increased lipid flux to the liver. Thus, impaired muscle protein turnover and autophagy may worsen MASLD indirectly by reducing muscle metabolic capacity and increasing dependence on hepatic lipid handling.

Mitochondrial dysfunction provides another link between skeletal muscle impairment and hepatic disease. In skeletal muscle, reduced mitochondrial oxidative capacity and impaired fatty acid oxidation limit substrate utilization, resulting in increased delivery of glucose and lipids to the liver. Sustained substrate overload overwhelms hepatic oxidative capacity, promoting lipid accumulation, ROS production, and disruption of mitochondrial quality-control pathways [119]. This establishes a shared mitochondrial dysfunction axis across tissues in which impaired muscle metabolism may amplify hepatic mitochondrial stress and disease progression.

Under conditions of overnutrition, inflammation, and physical inactivity, AMPK activity is suppressed, resulting in reduced fatty acid oxidation, impaired mitochondrial biogenesis, and increased lipogenesis [120,121]. In skeletal muscle, reduced AMPK signaling contributes to mitochondrial dysfunction and intramyocellular lipid accumulation, while in the liver, AMPK suppression promotes lipogenesis and impairs autophagy-mediated lipid degradation, accelerating progression from steatosis to hepatocellular injury [120]. In this setting, reduced AMPK activity represents one component of a broader metabolic failure, as skeletal muscle loses oxidative capacity and insulin responsiveness while the liver is exposed to greater lipid and glucose flux and worsening mitochondrial stress.

Disruption of myokine signaling represents an additional mechanistic link between muscle dysfunction and MASLD progression. In healthy states, exercise-induced myokines such as IL-6 and irisin regulate hepatic metabolism and mitochondrial function. In sarcopenic states, however, this signaling becomes dysregulated. IL-6 shifts from an acute metabolic regulator to a chronic low-grade inflammatory mediator, promoting proteolysis, insulin resistance, and systemic inflammation [122,123]. Reduced irisin signaling may further impair hepatic lipid oxidation and mitochondrial adaptation, particularly given experimental evidence linking irisin to AMPK-dependent mitochondrial regulation [124,125]. Alterations in adiponectin signaling exacerbate this process by reducing mitochondrial biogenesis and impairing autophagy-mediated quality control [126,127]. These changes reflect a broader loss of coordinated endocrine communication within the muscle–liver axis.

Systemic inflammation further reinforces muscle–liver axis dysfunction. Intramuscular lipid accumulation and insulin resistance promote the release of pro-inflammatory cytokines, including IL-6 and TNF-α, along with unfavorable adipokine profiles characterized by increased leptin and reduced adiponectin [110]. This inflammatory environment contributes to hepatic injury and fibrosis while simultaneously impairing muscle metabolism and protein homeostasis, thereby accelerating sarcopenia and reducing physical capacity. Reduced physical activity further exacerbates these processes by impairing mitochondrial function, limiting metabolic flexibility, and reducing the release of beneficial myokines. Clinically, the combination of sarcopenia and low physical activity is associated with increased risk of steatosis, fibrosis, and adverse outcomes [128].

Taken together, sarcopenia represents a critical disruption of the muscle–liver axis characterized by impaired substrate utilization, mitochondrial dysfunction, dysregulated myokine signaling, and chronic inflammation. These interconnected processes promote hepatic steatosis, inflammation, and fibrosis while further impairing skeletal muscle metabolism and function. Improving muscle function and metabolic signaling through physical activity may therefore reduce hepatic substrate burden, improve insulin sensitivity, and support more favorable muscle–liver communication in MASLD.

### 4.3. Exercise-Induced Hepatic Signaling via Myokines

Exercise improves the muscle–liver axis by increasing skeletal muscle substrate utilization and inducing muscle-derived endocrine signals that influence hepatic metabolism. During muscle contraction, skeletal muscle increases glucose and fatty acid utilization while releasing metabolites and myokines that regulate hepatic glucose production, lipid handling, and oxidative capacity [129]. In patients with MASLD, exercise improves hepatic steatosis, aminotransferases, and insulin sensitivity even in the absence of significant weight loss, highlighting its role as a primary therapeutic intervention [130]. Exercise-induced myokines and circulating factors also regulate hepatic transcriptional programs involved in lipid oxidation, mitochondrial respiration, and cellular stress responses [131]. Together, these findings suggest that exercise improves MASLD by reducing hepatic substrate overflow while activating muscle-derived signaling pathways that support hepatic mitochondrial adaptation.

Exercise induces myokine release through contraction-associated changes in oxidative stress, ROS signaling, ER stress, and transcriptional activation [129]. However, not all myokines have equal evidence linking them to exercise-mediated mitochondrial adaptation in MASLD. IL-6 and irisin currently have the strongest mechanistic support, whereas BAIBA, FGF21, adiponectin, extracellular vesicles, and microRNAs remain important but require further studies directly connecting exercise, MASLD, and hepatic mitochondrial remodeling.

Interleukin-6 (IL-6) is one of the most extensively studied exercise-induced myokines and coordinates hepatic glucose metabolism with mitochondrial energy demand. Circulating IL-6 levels increase in an intensity-dependent manner during exercise and can rise up to 100-fold during prolonged activity [132]. In hepatocytes, IL-6 activates STAT3 signaling pathways, promoting coordinated regulation of glucose production and utilization, with infusion studies demonstrating increased glucose appearance and disposal independent of insulin [133]. These effects are linked to AMPK activation through cAMP-dependent signaling that increases the AMP ratio and enhances substrate oxidation [122,134]. In MASLD, IL-6-mediated STAT3 activation may reduce substrate overload and de novo lipogenesis by suppressing lipogenic regulators, such as sterol regulatory element-binding protein 1 (SREBP1), while enhancing expression of fatty acid oxidation genes including CPT1 [132]. These exercise-induced effects are distinct from chronic inflammatory IL-6 signaling in metabolic disease, which suppresses mitochondrial function through stress-associated transcriptional pathways that reduce mitochondrial oxidative capacity [135]. Thus, transient exercise-induced IL-6 may support hepatic substrate mobilization and oxidation, whereas chronic inflammatory IL-6 contributes to mitochondrial dysfunction and insulin resistance [136].

Irisin, a PGC-1α-dependent myokine, has emerged as another important mediator of hepatic mitochondrial remodeling and bioenergetic adaptation [129]. In hepatocyte experiments and MASLD rodent models, irisin promotes mitochondrial fusion through upregulation of Mfn2 and Opa1, while suppressing excessive fission via reduced activation of Drp1, thereby enhancing mitochondrial network integrity [124,125]. Irisin also activates AMPK- and sirtuin-dependent signaling pathways, increasing expression of mitochondrial transcriptional regulators and enhancing electron transport chain activity [124,125]. These effects are associated with increased mitochondrial respiration and fatty acid oxidation, attenuation of oxidative and ER stress, reduced hepatic steatosis, improved insulin sensitivity, and restoration of mitochondrial function in MASLD models [124,125,137].

BAIBA primarily regulates hepatic metabolism through transcriptional control of fatty acid oxidation. BAIBA enhances hepatic β-oxidation via activation of PPARα signaling pathways, increasing expression of genes such as CPT1 and acyl-CoA oxidase while reducing lipogenesis and improving insulin sensitivity [138,139]. By activating PPARα-dependent transcription, BAIBA increases hepatic fatty acid import into mitochondria and peroxisomal β-oxidation, thereby reducing lipid accumulation and downstream lipotoxic stress. These effects are further supported by AMPK-dependent signaling that attenuates ER stress and improves metabolic homeostasis [140]. Although BAIBA does not appear to directly regulate mitochondrial biogenesis or dynamics, its ability to reduce lipid accumulation and metabolic stress may indirectly improve mitochondrial function by alleviating lipotoxicity and substrate overload.

Fibroblast growth factor-21 (FGF21) has emerged as an important endocrine regulator linking skeletal muscle, adipose tissue, and the liver during metabolic stress. Although the liver represents the principal source of circulating FGF21, skeletal muscle also produces FGF21 in response to exercise, mitochondrial stress, and energetic challenge. FGF21 promotes fatty acid oxidation, ketogenesis, glucose homeostasis, and mitochondrial oxidative capacity while suppressing hepatic lipogenesis and inflammation [141]. These actions make FGF21 an attractive therapeutic target in MASLD/MASH, as reflected by the development of several FGF21 analogues currently undergoing clinical investigation [142]. FGF21 may be most relevant in MASLD as a stress-responsive hepatokine and myokine that couples nutrient stress to adaptive lipid handling [143]. In exercise models, improved FGF21 responsiveness has been associated with reduced steatosis, enhanced insulin sensitivity, and activation of mitochondrial and autophagy programs that support lipid clearance [143]. FGF21 also regulates mitochondrial quality control through autophagy and mitophagy pathways, including epigenetic mechanisms that increase transcription of autophagy-related genes and facilitate lipid degradation and removal of damaged mitochondria [144]. In rodent models, restoration of FGF21 signaling reduces hepatic steatosis, improves insulin sensitivity, and enhances mitochondrial function, while exercise reverses deficits in FGF21 responsiveness associated with disease progression [145,146]. Together, these studies position FGF21 as an adaptive stress signal that may improve MASLD by linking exercise responsiveness to hepatic lipid clearance, insulin sensitivity, and mitochondrial quality control.

Paradoxically, circulating FGF21 concentrations are frequently elevated in patients with obesity and MASLD, suggesting the presence of a state of FGF21 resistance, analogous to insulin resistance [147,148]. Proposed mechanisms include reduced expression of the co-receptor β-Klotho, impaired FGFR1 signaling, chronic inflammatory activation, and persistent nutrient overload, resulting in diminished responsiveness despite elevated hormone concentrations. Exercise may partially overcome this resistance by reducing adiposity, improving insulin sensitivity, attenuating inflammation, and restoring mitochondrial function, thereby enhancing downstream responsiveness to endogenous FGF21 signaling. Although the precise mechanisms remain incompletely understood, reversal of FGF21 resistance represents another potential mechanism through which exercise contributes to hepatic mitochondrial reprogramming and metabolic improvement in MASLD/MASH.

Although adiponectin is primarily adipose-derived rather than muscle-derived, it is an exercise-responsive endocrine signal that integrates systemic insulin sensitivity, inflammation, and hepatic mitochondrial regulation [126,149,150]. It exerts direct effects on hepatic metabolism through AMPK- and sirtuin-dependent signaling pathways. In MASLD, adiponectin is most important as an insulin-sensitizing and anti-inflammatory signal that reduces hepatic lipotoxic stress. Its downstream effects on FAO, OXPHOS, and autophagy may therefore improve mitochondrial function both directly and by lowering the inflammatory and lipid burden placed on hepatocytes [126,149,150]. Adiponectin also reduces oxidative stress through mechanisms involving mitochondrial uncoupling, promotes AMPK-dependent autophagy, and facilitates clearance of damaged mitochondria [151]. In obesity and MASLD, reduced adiponectin signaling is associated with impaired mitochondrial function, increased inflammation, and disease progression, whereas restoration of adiponectin signaling improves hepatic steatosis, inflammation, and mitochondrial homeostasis [126].

Exercise also restores mitochondrial function and quality control across the muscle–liver axis. In skeletal muscle, exercise improves mitochondrial oxidative capacity and reduces oxidative stress through coordinated effects on mitochondrial biogenesis, autophagy/mitophagy, and fusion–fission dynamics [152]. These adaptations improve muscle substrate utilization and may reduce hepatic glucose and fatty acid overflow. In the liver, MASH-focused evidence suggests that exercise similarly improves mitochondrial redox balance and quality control, including biogenesis, mitophagy, and fusion–fission remodeling, thereby reducing ETC stress and mitochondrial ROS production [22]. In MASLD, where mitochondrial dysfunction contributes to impaired fatty acid oxidation and oxidative stress, these changes improve hepatic lipid handling and reduce susceptibility to hepatocellular injury. Within the setting of sarcopenia, exercise improves skeletal muscle mass, strength, and metabolic function while simultaneously reducing hepatic steatosis and inflammation [111,153]. Enhanced muscle insulin sensitivity and oxidative capacity limit excess substrate delivery to the liver, while restoration of endocrine signaling supports hepatic metabolic adaptation.

Emerging experimental evidence also suggests muscle-derived extracellular vesicles and microRNAs may contribute to exercise-induced muscle–liver communication by altering hepatic gene expression and systemic metabolic regulation [154,155]. These findings suggest extracellular vesicle signaling may represent an additional mechanism through which exercise links skeletal muscle activity to hepatic metabolic adaptation, although its role in MASLD/MASH and mitochondrial remodeling remains incompletely defined.

**Figure 2:**
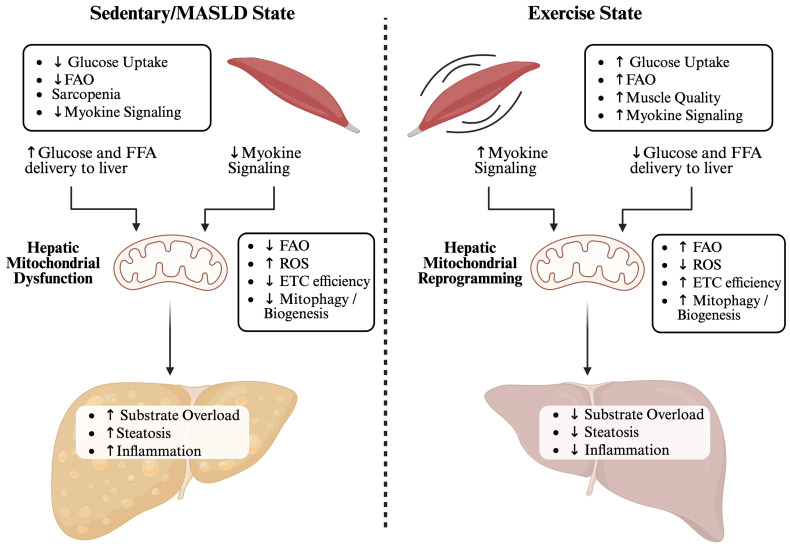
Exercise-Induced Mitochondrial Reprogramming of the Muscle–Liver Axis in MASLD. ETC: Electron Transport Chain; FAO: Fatty Acid Oxidation; FFA: Free Fatty Acid; ROS: Reactive Oxygen Species. Created in BioRender. Jonas McCaffrey (2026). https://BioRender.com/5otxori (accessed on 4 June 2026).

Overall, exercise-induced myokines and related endocrine signals regulate hepatic metabolism through effects on glucose handling, lipid metabolism, and mitochondrial function. Current evidence most strongly supports IL-6 and irisin as exercise-responsive mediators of hepatic metabolic and mitochondrial adaptation, while BAIBA, FGF21, adiponectin, extracellular vesicles, and microRNAs remain promising but less directly established in the specific context of exercise-induced mitochondrial remodeling in MASLD/MASH. These signals help explain how improved skeletal muscle activity can translate into reduced hepatic lipid accumulation, mitochondrial stress, and inflammation in MASLD. A summary of exercise-induced mitochondrial reprogramming of the muscle-liver axis is depicted in Figure 2. 

Collectively, skeletal muscle should be viewed not simply as an organ of locomotion but as a dynamic endocrine organ that orchestrates systemic metabolic adaptation during exercise. Through coordinated release of myokines, improved substrate partitioning, enhanced insulin sensitivity, and endocrine signaling pathways including FGF21, skeletal muscle communicates directly with the liver to regulate mitochondrial energetics, inflammatory signaling, and lipid metabolism. These integrated responses converge upon hepatic mitochondrial reprogramming, providing a mechanistic explanation for how exercise exerts beneficial hepatic effects that are disproportionate to changes in body weight alone.

## 5. Gut–Liver Crosstalk in MASLD and Exercise

The gut–liver axis links intestinal microbes, barrier integrity, bile acids, and microbial metabolites with hepatic immune and metabolic signaling. In MASLD, dysbiosis and increased intestinal permeability increase hepatic exposure to inflammatory microbial products, contributing to lipid accumulation, immune activation, and mitochondrial stress. Exercise may counter these processes by improving microbial ecology, barrier function, and metabolite signaling.

### 5.1. Normal Gut–Liver Axis Signaling

The gut–liver axis is a bidirectional communication network linking the gastrointestinal tract and liver through vascular, metabolic, and immunologic pathways. This relationship is primarily established through the portal circulation, which delivers gut-derived nutrients, microbial products, and metabolites directly to the liver, enabling first-pass hepatic exposure to intestinal contents. This anatomical arrangement positions the liver as a central immunometabolic interface between the external environment and systemic circulation [156].

The biliary system reinforces this bidirectional signaling. Through secretion of bile acids, immunoglobulins, and antimicrobial peptides into the intestinal lumen, the liver helps regulate microbial composition, mucosal immune activity, and intestinal metabolic signaling [156]. In turn, microbial metabolism modifies bile acid pools and generates metabolites that return to the liver through portal circulation. This reciprocal signaling allows the gut microbiome and liver to coordinate hepatic lipid handling, inflammatory tone, and metabolic adaptation.

A critical component of this axis is the intestinal barrier, which limits systemic exposure to luminal microbes and toxins while permitting selective nutrient absorption. The barrier includes epithelial tight junction proteins, the mucus layer, vascular components, and immune defenses [157]. Tight junction proteins including occludin and claudins regulate paracellular permeability, while Paneth cell-derived antimicrobial peptides help regulate microbial populations and prevent bacterial overgrowth. Together, these mechanisms maintain intestinal integrity and limit translocation of microbial products into portal circulation [158]. In this way, the intestinal barrier functions as a gatekeeper of hepatic mitochondrial stress by regulating the magnitude of microbial and inflammatory signals delivered to the liver.

Gut microbiota are essential mediators of gut–liver signaling and contribute to host metabolism, immune regulation, and nutrient processing [159]. In healthy individuals, microbial communities are dominated by Firmicutes and Bacteroidetes, along with less abundant phyla involved in energy harvest, vitamin synthesis, and immune development [159,160]. Microbial metabolites, particularly short-chain fatty acids (SCFAs) and bile acids, provide major signaling links between the intestine and liver. SCFAs, including acetate, propionate, and butyrate, are produced through bacterial fermentation of dietary substrates and regulate metabolic and immune pathways through G protein-coupled receptor signaling and epigenetic mechanisms [161]. SCFAs also support intestinal barrier integrity and anti-inflammatory immune responses while bile acids signal through receptors such as farnesoid X receptor (FXR) and TGR5 to influence hepatic and intestinal metabolism [161,162].

Immune signaling provides another key connection between gut-derived inputs and hepatic function. Pattern recognition receptors, including Toll-like receptors (TLRs), detect microbial products such as lipopolysaccharide (LPS) and bacterial DNA, activating downstream inflammatory pathways within the liver [163,164]. When barrier integrity is compromised, increased delivery of microbial ligands can shift this response toward Kupffer cell activation, inflammatory cytokine production, and hepatocellular injury [156]. Thus, gut-derived immune signals directly influence the inflammatory environment in which hepatic mitochondrial dysfunction and lipid stress develop.

Together, the gut–liver axis maintains hepatic metabolic homeostasis by regulating microbial composition, barrier integrity, bile acid signaling, SCFA production, and immune activation. This framework is central to MASLD/MASH because disruption of these pathways can increase portal inflammatory signals, alter hepatic lipid handling, and contribute to mitochondrial stress. Normal gut–liver signaling therefore provides the physiologic baseline for understanding how dysbiosis and impaired barrier function promote MASLD progression, and how exercise may restore more favorable gut-derived metabolic and inflammatory inputs to the liver.

Although changes in gut microbial composition have received considerable attention, increasing evidence suggests that alterations in microbial metabolic function may be even more relevant to host physiology. Exercise influences numerous microbial pathways involved in bile acid metabolism, SCFA biosynthesis, branched-chain amino acid (BCAA) metabolism, tryptophan metabolism, and choline utilization, all of which directly influence hepatic mitochondrial function.

Exercise-induced modifications of bile acid metabolism alter activation of the farnesoid X receptor (FXR) and the G protein-coupled bile acid receptor TGR5. Activation of these signaling pathways regulates hepatic lipid metabolism, glucose homeostasis, mitochondrial oxidative capacity, and inflammatory responses while simultaneously influencing energy expenditure through endocrine communication between the intestine and liver. Similarly, exercise-induced reductions in microbial production of potentially harmful metabolites, including trimethylamine (TMA) and its hepatic product trimethylamine N-oxide (TMAO), may reduce inflammatory signaling and metabolic dysfunction [165].

Exercise also remodels microbial amino acid metabolism, including branched-chain amino acid and tryptophan pathways. Elevated circulating BCAAs have been associated with insulin resistance and MASLD, whereas exercise appears to improve BCAA utilization through enhanced skeletal muscle oxidation. In parallel, exercise modifies microbial tryptophan metabolism, influencing production of indole derivatives that activate the aryl hydrocarbon receptor and contribute to intestinal barrier integrity, immune regulation, and hepatic metabolic homeostasis [166]. Collectively, these observations suggest that exercise influences not only microbial composition but also microbial metabolic function, thereby providing an additional mechanism through which exercise promotes hepatic mitochondrial reprogramming.

Although numerous studies report increased microbial production of SCFAs following regular exercise, findings have not been entirely consistent [167]. Variability likely reflects differences in exercise modality, training duration, dietary composition, baseline microbiome characteristics, obesity status, and analytical methodologies used to quantify microbial metabolites. Furthermore, individual SCFAs exert distinct physiological effects that may differ according to tissue distribution and metabolic context. Consequently, current evidence supports a beneficial overall effect of exercise on microbial metabolism, while emphasizing that specific alterations in SCFA production remain influenced by host and environmental factors. Recognition of these complexities underscores the importance of integrating microbial functional analyses with traditional taxonomic profiling in future exercise studies.

### 5.2. Gut Microbiome Dysfunction in MASLD

Alterations in the gut microbiome contribute to MASLD pathogenesis not only through changes in microbial composition but also through disruption of microbial metabolic functions that regulate host metabolism, immune signaling, and intestinal barrier integrity. Dysbiosis in MASLD is characterized by reduced microbial diversity and expansion of pathogenic taxa, with these changes correlating with disease severity and progression [168,169]. Patients with MASLD exhibit decreased α-diversity and shifts in microbial composition, including depletion of beneficial commensal species such as *Faecalibacterium, Ruminococcus,* and *Coprococcus,* alongside enrichment of potentially pathogenic taxa including *Escherichia*, *Streptococcus*, and *Prevotella* [168,169]. These compositional changes are accompanied by progressive loss of microbial stability and increased abundance of pathobionts with advancing disease, suggesting a transition toward a more pro-inflammatory microbial environment [168].

Beyond taxonomic alterations, functional changes in microbial metabolism contribute to disease progression. The gut microbiome produces metabolites including short-chain fatty acids (SCFAs), bile acids, ethanol, and trimethylamine N-oxide (TMAO), which influence hepatic lipid metabolism, insulin sensitivity, inflammatory signaling, oxidative stress, and fibrogenesis [168,170]. These metabolites provide a mechanistic link between dysbiosis and hepatic mitochondrial stress by altering substrate handling, inflammatory tone, and redox balance.

Endogenously produced microbial ethanol has emerged as an important contributor to MASLD. Patients with MASH exhibit elevated circulating ethanol levels that increase with disease severity, promoting hepatocellular injury and inflammatory signaling [168]. In a prospective clinical study, portal vein ethanol concentrations were substantially higher than peripheral blood levels and increased across disease stages, while antibiotic-sensitive changes in peripheral ethanol supported a microbial origin [171]. High-alcohol-producing Klebsiella pneumoniae has also been associated with MASLD in human cohorts, and colonization or fecal microbiota transfer from a patient harboring this strain induced MASLD in mice, whereas selective elimination prevented disease [172]. Mechanistically, endogenous ethanol metabolism generates acetaldehyde and ROS, while ethanol and acetaldehyde increase intestinal permeability, promoting LPS translocation, Kupffer cell activation, and inflammatory cytokine release. Experimental models further support causality, as high-ethanol-producing Klebsiella strains induce steatosis, mitochondrial ROS accumulation, DNA damage, and ATP depletion, whereas alcohol dehydrogenase-deficient strains produce less pathology [163].

Bile acid metabolism is also significantly altered in MASLD. The gut microbiota regulates bile acid composition through enzymatic conversion into secondary bile acids, which signal through receptors such as FXR and TGR5 [162]. Disruption of this process alters bile acid pools and impairs pathways regulating hepatic lipid metabolism, bile acid synthesis, and glucose homeostasis. A meta-analysis of biopsy-proven human studies showed that circulating bile acids are elevated in MASLD, with total bile acid levels higher than in healthy controls and conjugated bile acid species showing potential ability to distinguish MASH from simple steatosis [173]. Advanced fibrosis has been associated with higher total plasma bile acids, increased glyco- and tauro-conjugated chenodeoxycholic acid family bile acids, and higher unconjugated lithocholic acid [174]. Mechanistically, dysbiosis can alter conjugated and unconjugated bile acid pools and disrupt FXR/TGR5 signaling. In experimental models, this increases CYP7A1 and SREBP-1c, promoting bile acid synthesis, lipogenesis, cholestasis, and steatosis [175]. Increased levels of certain secondary bile acids, including lithocholic acid species, have also been associated with hepatotoxicity and fibrosis progression [168,176].

SCFAs represent another important interface between the gut microbiome and host metabolism. In MASLD, depletion of SCFA-producing bacteria such as *Bifidobacterium, Faecalibacterium Prausnitzii,* and *Ruminococcus* are accompanied by altered SCFA availability and distribution [168,169]. Meta-analyses and biopsy-proven MASLD cohorts support reduced abundance of *Ruminococcus*, *Faecalibacterium*, and *Coprococcus* in MASLD or MASH, although findings for Bifidobacterium are less consistent [177,178]. SCFA findings are compartment- and stage-dependent: stool SCFAs may be reduced in some MASLD cohorts, whereas fecal or circulating SCFAs may be increased or associated with disease phenotype in others, suggesting altered production, absorption, permeability, or hepatic clearance rather than a simple deficiency state [179].

Loss of beneficial SCFA signaling may impair intestinal barrier integrity and reduce anti-inflammatory signaling, contributing to increased intestinal permeability, metabolic dysregulation, insulin resistance, and hepatic lipid accumulation [179]. In animal models, sodium butyrate attenuated diet-induced steatohepatitis by restoring gut microbial balance, improving tight junction integrity, reducing portal endotoxin exposure, and suppressing hepatic inflammatory signaling [180]. These findings support a protective role for butyrate in MASLD by linking improved barrier integrity with reduced portal endotoxin exposure, lower hepatic inflammation, and decreased mitochondrial stress [181].

One mechanistic link between dysbiosis and liver injury is disruption of the intestinal barrier. Increased intestinal permeability permits translocation of bacterial products such as lipopolysaccharide (LPS) into portal circulation, where they activate hepatic immune pathways [168]. Activation of Toll-like receptor 4 (TLR4) signaling promotes inflammatory cascades, including TNF-α production and inflammasome activation, leading to hepatocyte injury, stellate cell activation, and fibrogenesis. Although clinical data on circulating LPS levels are variable, the association between increased gut permeability and MASLD is well supported [182]. Thus, barrier dysfunction provides a direct route by which microbial signals amplify hepatic inflammation, lipotoxicity, and mitochondrial stress.

Microbial alterations in MASLD extend beyond bacteria to include the gut mycobiome and virome. Reduced fungal diversity and increased abundance of species such as Candida albicans have been associated with advanced fibrosis, while changes in the intestinal virome have also been linked to disease severity [168]. Multiple microbial domains may therefore contribute to MASLD pathogenesis beyond bacterial dysbiosis alone. Given the role of dysbiosis, microbial metabolites, and intestinal barrier dysfunction in MASLD progression, exercise may modify gut–liver crosstalk by reshaping microbial composition, restoring barrier integrity, and altering metabolite signaling.

### 5.3. Effects of Exercise on Gut–Liver Crosstalk in MASLD

Exercise may improve gut–liver crosstalk in MASLD by reducing gut-derived inflammatory inputs and restoring microbial metabolite pathways that regulate hepatic lipid metabolism. These adaptations are relevant because dysbiosis, impaired bile acid signaling, and increased intestinal permeability promote hepatic immune activation, steatosis, and mitochondrial stress [183]. Within the broader framework of this review, exercise-induced gut–liver remodeling represents one component of a systems-level mitochondrial adaptation in which microbial, inflammatory, and metabolite signals converge on hepatic β-oxidation, mitochondrial ROS production, and mitochondrial quality control.

At the level of the gut microbiome, exercise interventions remodel microbial communities in both human and animal models of MASLD. In a randomized controlled trial of patients with MASLD and prediabetes, exercise and diet interventions increased microbial network connectivity and robustness, suggesting improved ecosystem stability, while combined aerobic exercise and diet diversified and stabilized keystone taxa [184]. Baseline gut microbial network structure predicted the degree of liver fat reduction in response to intervention, suggesting microbiome composition may influence therapeutic responsiveness [184]. A study in biopsy-proven MASH patients similarly demonstrated exercise could reverse gut dysbiosis in established disease, shifting the microbial profile toward that of healthy controls [185]. In insulin-resistant subjects, both sprint interval training and moderate-intensity continuous training increased *Bacteroidetes*, decreased the *Firmicutes/Bacteroidetes* ratio, and reduced systemic and intestinal inflammatory markers including TNF-α and LPS-binding protein [186]. Although the *Firmicutes/Bacteroidetes* ratio is a variable marker of obesity-associated dysbiosis, these changes suggest a shift away from a metabolically inflammatory microbial profile. In preclinical models, exercise increases microbial diversity and promotes enrichment of beneficial taxa, including *Bacteroidetes* and SCFA-producing bacteria, while reducing Proteobacteria, a phylum enriched in Gram-negative pathobionts linked to endotoxin-mediated inflammation [187,188]. These studies suggest that exercise responsiveness in MASLD may depend partly on baseline microbial community structure and the capacity of exercise to restore a more stable, metabolically favorable microbial network. Importantly, this remodeling is relevant to mitochondrial adaptation because reduced pathobiont expansion and lower inflammatory microbial signaling may decrease portal immune activation, thereby limiting cytokine-driven mitochondrial ROS production and hepatic oxidative injury.

Exercise also improves intestinal barrier function, an important determinant of gut-derived inflammatory signaling in MASLD. Increased intestinal permeability permits translocation of bacterial products into portal circulation, promoting hepatic immune activation and disruption of lipid metabolism [183]. In experimental studies, aerobic exercise restored tight junction protein expression and reduced gut-derived LPS exposure, consistent with decreased bacterial translocation [189]. In HFD-induced MASH mice, treadmill exercise reduced serum LPS and hepatic inflammatory signaling while alleviating hepatic lipid accumulation, inflammation, and fibrosis [190]. These findings suggest improved barrier integrity may reduce hepatic exposure to endotoxin-driven inflammation and secondarily lessen mitochondrial ROS production and hepatocellular stress. Although acute exercise may transiently increase intestinal permeability, chronic training appears to promote adaptive improvements in epithelial defense and barrier integrity [191]. Thus, exercise-mediated barrier restoration may protect hepatic mitochondria by lowering the inflammatory stimuli that impair electron transport, amplify oxidative stress, and promote lipotoxic injury.

Alterations in microbial metabolites may mediate exercise-induced gut–liver signaling. Exercise increases SCFA production in animal models, in some cases exceeding the effects of fiber supplementation alone [188]. These changes are accompanied by enrichment of butyrate-producing taxa and improvements in hepatic steatosis and lipid metabolism [188,189]. In a rat model of MASLD, aerobic exercise upregulated butyrate-producing bacterial taxa, bile acid-related gene expression, and intestinal barrier function, with higher-load exercise producing stronger effects on lipid metabolism [187]. SCFAs, particularly butyrate, may benefit hepatic metabolism by strengthening epithelial barrier integrity, reducing endotoxin exposure, and limiting inflammatory signaling. In experimental models, these effects are associated with improved lipid oxidation, reduced de novo lipogenesis, and lower hepatic inflammatory burden [192]. Thus, exercise-induced enrichment of SCFA-producing bacteria may reduce hepatic mitochondrial stress indirectly by strengthening barrier integrity, lowering endotoxin exposure, and decreasing inflammatory and lipotoxic inputs to the liver. In this way, SCFA-related remodeling links gut microbial adaptation to mitochondrial protection by reducing the substrate overload and inflammatory tone that otherwise impair β-oxidation and increase ROS generation.

Exercise also impacts bile acid metabolism, with both human and animal studies demonstrating changes in bile acid pools and bile acid-related gene expression [187,193]. In a 12-week HIIT trial in patients with MASLD, glyco-conjugated bile acids decreased after exercise alongside improvements in fasting glucose and waist circumference [193]. These findings suggest that bile acid remodeling may be one mechanism linking exercise to improved hepatic and systemic metabolic regulation. Because dysbiosis in MASLD impairs microbial bile acid metabolism and signaling through receptors such as FXR and TGR5, bile acid pathways represent a plausible link between gut microbiome changes and hepatic lipid and glucose regulation [183,192]. Exercise-associated bile acid remodeling may therefore reduce hepatic lipogenesis, improve glucose regulation, and indirectly protect mitochondrial function by limiting lipid overload and inflammatory stress. Although direct evidence regarding exercise-mediated reductions in endogenous ethanol and TMAO in MASLD remains limited, reductions in pathogenic taxa and improved barrier integrity suggest potential downstream effects on these metabolite pathways [194].

Emerging evidence further suggests that exercise-dependent gut–liver signaling intersects with hepatic mitochondrial biology. HFD-induced MASLD is associated with mitochondrial and endoplasmic reticulum oxidative stress, which promotes lipogenesis and hepatic lipid accumulation [183]. In preclinical MASLD models, exercise has been shown to improve mitochondrial function by enhancing β-oxidation, increasing electron transport chain efficiency, reducing ROS production, and restoring mitochondrial quality control [22]. In MASLD mice, voluntary wheel running improved hepatic mitochondrial function, optimized the mitochondrial unfolded protein response, and increased FGF21 secretion, linking exercise-induced mitochondrial adaptation to hepatoprotective signaling [145]. Exercise-induced skeletal muscle FGF21 has also been shown to promote hepatic lipophagy through an AMPK-dependent pathway, linking inter-organ crosstalk to hepatic lipid clearance [194].

These mitochondrial effects may be partly mediated by reduced gut-derived inflammatory stress and improved microbial metabolite signaling. By reducing endotoxin exposure and improving SCFA and bile acid pathways, exercise may lessen hepatic ROS generation while supporting β-oxidation, electron transport chain efficiency, antioxidant defenses, and mitochondrial quality control. FGF21 may also link hepatic metabolic stress responses to microbiome remodeling, as exercise-induced FGF21 increased the abundance of beneficial gut microbes in experimental models, while FGF21 knockout or microbiome disruption reduced the hepatoprotective effects of exercise [195]. Exercise-induced AMPK activation as well as Sestrin2 (SESN2) activation may further integrate these responses by improving barrier integrity, promoting lipophagy, enhancing antioxidant defenses, and supporting mitochondrial quality control under metabolic stress [196,197]. Together, these pathways suggest that exercise-mediated gut–liver remodeling supports mitochondrial adaptation not only by changing microbial composition, but by reducing inflammatory pressure and improving the metabolic signals that regulate hepatic lipid oxidation and organelle quality control.

**Figure 3:**
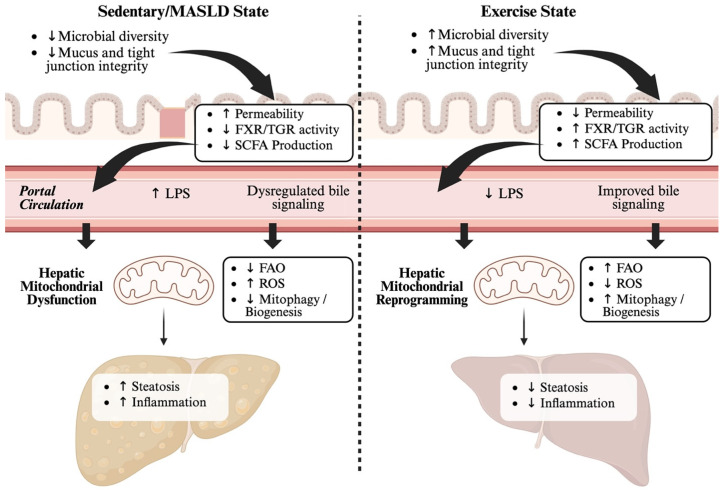
Exercise-Induced Mitochondrial Reprogramming of the Gut–Liver Axis in MASLD. FAO: Fatty Acid Oxidation; FXR: Farnesoid X Receptor; LPS: Lipopolysaccharide; ROS: Reactive Oxygen Species; SCFA: Short-Chain Fatty Acid. Created in BioRender.com. Jonas McCaffrey (2026). https://BioRender.com/99oqaxp (accessed on 4 June 2026).

Exercise appears to improve gut–liver crosstalk primarily by reducing hepatic inflammatory and metabolic burden. By strengthening barrier integrity, increasing beneficial microbial taxa, improving SCFA production, and altering bile acid signaling, exercise may decrease gut-derived inputs that amplify hepatic steatosis, inflammation, and mitochondrial stress. However, evidence linking exercise-induced microbiome changes to hepatic mitochondrial remodeling in human MASLD remains limited, making this an important area for future mechanistic studies. Overall, exercise-induced microbiome remodeling should be viewed not as an isolated gut effect, but as a mechanism that may reduce mitochondrial inflammatory stress, improve hepatic substrate handling, and support mitochondrial resilience in MASLD/MASH. A schematic of mitochondrial specific of effects of exercise on the gut-liver axis in MASLD is depicted in Figure 3.

## 6. Thyroid–Liver Crosstalk in MASLD and Exercise

Thyroid hormone signaling is a major regulator of hepatic lipid metabolism, energy expenditure, and mitochondrial function. Altered systemic and intrahepatic thyroid signaling is associated with MASLD progression, while exercise may influence thyroid–liver crosstalk through context-dependent changes in circulating thyroid hormones, deiodinase activity, and local T3/TRβ-mediated mitochondrial adaptation.

### 6.1. Normal Thyroid Hormone Signaling in the Liver

Thyroid hormone signaling regulates hepatic energy metabolism by coordinating lipid turnover, glucose handling, oxygen consumption, and mitochondrial oxidative capacity. The thyroid gland predominantly secretes thyroxine (T4), which functions as a prohormone and is converted to the biologically active triiodothyronine (T3) through tissue-specific deiodinase activity [198]. Alternatively, T4 can be converted to reverse T3 (rT3), an inactive thyroid hormone metabolite that does not activate thyroid hormone receptors and therefore reduces effective thyroid hormone signaling. The liver represents a primary target organ for thyroid hormone action, where signaling coordinates lipid, glucose, and mitochondrial metabolism [199].

Deiodinases are selenoprotein enzymes that modulate intracellular thyroid hormone availability through three isoforms [198]. Type 1 (D1) and type 2 (D2) deiodinases catalyze activation of T4 to T3, whereas type 3 deiodinase (D3) inactivates T3 and T4, thereby attenuating signaling [200]. D1 is highly expressed in the liver and kidney, where it contributes to circulating T3 levels and iodine recycling, while D2 is expressed in metabolically active tissues including skeletal muscle and adipose tissue and contributes to local metabolic regulation [199]. This tissue-specific control of T3 availability is important because circulating thyroid hormone concentrations may not fully reflect local thyroid hormone action in metabolically active tissues.

Thyroid hormone effects are mediated through nuclear thyroid hormone receptors (TRs), encoded by THRA and THRB, which generate multiple isoforms with distinct tissue distributions [201]. TRα predominates in skeletal muscle, cardiac tissue, and the central nervous system, whereas TRβ is the dominant isoform in the liver, with TRβ1 serving as the principal mediator of hepatic thyroid hormone signaling. Activation of TRβ1 regulates hepatic pathways involved in lipid metabolism, cholesterol turnover, gluconeogenesis, and insulin sensitivity [199].

At the molecular level, thyroid hormone signaling occurs through both genomic and non-genomic mechanisms [202]. Genomically, T3 binds nuclear TRs, which act as ligand-dependent transcription factors to regulate gene expression [203]. Ligand binding induces recruitment of coactivators and activation of transcriptional programs involved in lipid metabolism, mitochondrial function, and energy homeostasis. Non-genomic pathways allow more rapid cellular responses independent of direct DNA binding, including activation of membrane-associated receptors such as integrin αvβ3 and intracellular signaling cascades involving PI3K/Akt and MAPK pathways [203]. These pathways regulate cellular metabolism and kinase signaling and can converge with genomic signaling to influence transcriptional outcomes [202].

Thyroid hormones exert profound effects on mitochondrial biology through which regulation of cellular energetics can occur [204]. T3 stimulates mitochondrial biogenesis through coordinated transcriptional activation of nuclear and mitochondrial regulators, including PGC-1α, NRF-1, and NRF-2 [205]. These factors promote expression of genes involved in OXPHOS, the TCA cycle, and fatty acid β-oxidation, leading to increased mitochondrial capacity and ATP production [205]. T3 also regulates mitochondrial turnover through modulation of fission and mitophagy pathways and can rapidly enhance mitochondrial respiration through non-genomic mechanisms that increase ATP production and metabolic flux [204,206]. In hepatocytes, these effects support lipid disposal and metabolic flexibility, making thyroid hormone signaling directly relevant to mitochondrial vulnerability in MASLD [204,205].

In the liver, thyroid hormone plays a critical role in coordinating lipid, cholesterol, and glucose metabolism. T3 stimulates both DNL and fatty acid β-oxidation, reflecting its role in maintaining metabolic flexibility [207]. Lipogenic effects are mediated in part through activation of carbohydrate-responsive element-binding protein (ChREBP), whereas fatty acid oxidation is enhanced through mitochondrial mechanisms involving increased fatty acid transport into mitochondria, activation of β-oxidation enzymes, improved respiratory chain activity, and PGC-1α-dependent mitochondrial biogenesis [207]. This dual regulation supports dynamic control of hepatic TG turnover. Thyroid hormone also regulates cholesterol metabolism, with hypothyroidism associated with increased LDL cholesterol and triglyceride levels, and hyperthyroidism promoting enhanced lipoprotein clearance [207]. Thyroid hormone regulates energy expenditure and glucose homeostasis through effects on ATP turnover and substrate cycling [201]. In the liver, T3 also modulates gluconeogenesis and insulin sensitivity, linking thyroid hormone activity to broader control of hepatic glucose flux [199]. Together, these effects allow thyroid hormone to increase hepatic lipid and glucose turnover while maintaining oxidative capacity, making impaired thyroid hormone signaling particularly relevant to MASLD.

Overall, thyroid hormone signaling integrates receptor-mediated transcriptional regulation, rapid kinase signaling, and mitochondrial adaptation to coordinate hepatic energy metabolism. These effects position thyroid hormone signaling as an important regulator of liver metabolic homeostasis and provide the basis for understanding how altered thyroid signaling may contribute to MASLD [208].

Beyond its classical effects on basal metabolic rate, thyroid hormone functions as a master regulator of mitochondrial biology. T3 stimulates transcription of nuclear genes encoding mitochondrial proteins, enhances mitochondrial biogenesis through activation of PGC-1α and nuclear respiratory factors, promotes fatty acid oxidation, increases oxidative phosphorylation, and accelerates mitochondrial turnover through coordinated regulation of fusion, fission, and mitophagy [199]. Consequently, thyroid hormone signaling influences virtually every component of hepatic mitochondrial reprogramming, including mitochondrial energetics, quality control, substrate utilization, and redox homeostasis.

Exercise and thyroid hormone signaling exhibit substantial mechanistic overlap. Both activate AMPK–PGC-1α pathways, enhance mitochondrial oxidative capacity, improve metabolic flexibility, and suppress hepatic lipogenesis. Rather than functioning as independent regulators, these pathways likely operate synergistically to optimize mitochondrial performance during increased energetic demand. This mechanistic convergence provides a biological rationale for combining structured exercise with therapeutic modulation of thyroid hormone signaling in patients with MASLD/MASH.

### 6.2. Thyroid Dysfunction in MASLD

Thyroid dysfunction is consistently associated with MASLD development and progression. In a large meta-analysis including approximately 76.5 million individuals, primary hypothyroidism was associated with increased MASLD prevalence and higher risk of MASH or advanced fibrosis, while cohort data showed a 1.71-fold increased MASLD risk in hypothyroid individuals compared with euthyroid controls [209]. Additional meta-analytic data support this association, linking hypothyroidism to nearly a twofold increased risk of MASLD/MASH and identifying subclinical hypothyroidism as a risk factor for MASLD prevalence and advanced fibrosis [210]. Even within the euthyroid range, higher TSH levels are associated with a 41–72% increased risk of advanced fibrosis depending on the scoring method used [211]. This relationship may be bidirectional, as individuals with MASLD are more likely to develop hypothyroidism, mainly subclinical disease [210].

In addition to systemic hypothyroidism, MASLD is associated with intrahepatic thyroid hormone dysregulation. Patients with MASH may develop a state of “intrahepatic hypothyroidism,” characterized by reduced conversion of T4 to active T3 and increased conversion of T4 to reverse T3, resulting in impaired intrahepatic T3/TRβ signaling despite normal circulating thyroid hormone levels [212]. This localized reduction in thyroid hormone activity may limit TRβ-dependent transcriptional programs that support hepatic fatty acid oxidation, mitochondrial biogenesis, and metabolic homeostasis. Clinically, reduced free T3 has been associated with advanced fibrosis and increased liver stiffness in patients at high risk for NASH, although low fT3 may also reflect more advanced systemic or hepatic illness rather than a direct causal mechanism [213]. Altered central sensitivity to thyroid hormones has also been associated with MASLD prevalence and fibrosis risk, suggesting that tissue-level thyroid hormone responsiveness may be relevant even when standard circulating thyroid hormone levels are preserved [214]. These findings suggest that impaired systemic, intrahepatic, and tissue-level thyroid hormone signaling may converge to reduce hepatic lipid disposal and mitochondrial adaptability in MASLD/MASH.

Disruption of thyroid hormone signaling promotes hepatic steatosis through effects on lipid metabolism, insulin sensitivity, and lipid turnover. In hypothyroid states, reduced insulin sensitivity and impaired suppression of adipose tissue lipolysis increase circulating FFAs, enhancing hepatic lipid delivery and TG accumulation [215]. Concurrently, decreased hepatic fatty acid β-oxidation and increased DNL, driven by hyperglycemia and activation of lipogenic transcriptional pathways including ChREBP, promote lipid retention and lipotoxicity [207,215]. Altered cholesterol metabolism, including reduced hepatic LDL receptor expression, contributes to increased circulating LDL cholesterol, triglycerides, and apolipoprotein B. Impairment of autophagy and lipophagy further limits lipid droplet degradation, exacerbating triglyceride accumulation [207]. These mechanisms increase hepatic lipid delivery while reducing lipid disposal, creating conditions that favor steatosis and lipotoxic stress.

Mitochondrial dysfunction links thyroid hormone deficiency to MASLD progression as well. As previously mentioned, T3 promotes mitochondrial biogenesis and function through transcriptional regulation of mediators such as PGC-1α and nuclear respiratory factors. In hypothyroid states, this coordinated program is disrupted, resulting in reduced mitochondrial content, impaired OXPHOS, and diminished fatty acid oxidation capacity [208]. Loss of thyroid hormone signaling also impairs mitochondrial substrate utilization and electron transport chain activity, leading to accumulation of lipotoxic intermediates and increased ROS production. Disruption of mitochondrial calcium handling further compromises ATP production and metabolic efficiency [208]. These mitochondrial defects may promote oxidative stress and hepatocellular injury, thereby supporting progression from steatosis to MASH.

Thyroid dysfunction also worsens systemic glucose metabolism and energy expenditure. In experimental models of mild hypothyroidism, impaired adipose tissue insulin sensitivity and reduced insulin secretion increase fatty acid flux to the liver; the resulting hepatic lipid accumulation induces hepatic insulin resistance and impaired suppression of endogenous glucose production, which can further stimulate de novo lipogenesis and promote steatosis [215]. Clinical cohort data support an association between hypothyroidism and incident MASLD, with mediation analyses suggesting that metabolic and inflammatory intermediaries may partly explain this relationship, although the relative contribution of individual markers such as HbA1c remains incompletely defined [209]. At the systemic level, hypothyroidism is associated with a hypometabolic state characterized by reduced energy expenditure, impaired lipolysis, and weight gain, which further promote hepatic lipid accumulation and disease progression [216].

These metabolic and mitochondrial disturbances are associated with adverse clinical outcomes. Subclinical hypothyroidism has been linked to increased all-cause and cardiovascular mortality in MASLD populations [217]. Thyroid dysfunction is also associated with accelerated fibrosis progression, reflected by higher FIB-4 scores and increased prevalence of advanced steatosis [218].

Therapeutic targeting of hepatic thyroid hormone signaling further supports its relevance in MASLD. Resmetirom, a liver-directed TRβ-selective agonist, received FDA approval in 2024 for the treatment of noncirrhotic MASH with moderate to advanced fibrosis [153]. In the phase 3 MAESTRO-MASH trial, resmetirom significantly improved histologic outcomes, with MASH resolution observed in approximately 26–30% of treated patients compared with 9.7% in the placebo group, and fibrosis improvement in approximately 24–26% versus 14.2% in controls [219]. These effects were accompanied by reductions in lipids, including LDL cholesterol and triglycerides [219]. Mechanistically, resmetirom activates hepatic TRβ signaling, enhancing mitochondrial fatty acid oxidation, improving lipid handling, and promoting mitochondrial biogenesis [212]. These findings support thyroid hormone signaling as a clinically relevant regulator of hepatic lipid metabolism, mitochondrial function, and MASLD progression.

### 6.3. Effects of Exercise on Thyroid–Liver Crosstalk in MASLD

Exercise induces dynamic and context-dependent alterations in thyroid hormone signaling that reflect both acute metabolic stress and chronic adaptation. Acute exercise produces biphasic changes in circulating thyroid hormones, characterized by transient increases in T3 and T4 followed by reductions in T3 and a shift toward reverse T3 with prolonged exertion or energy deficit. This is consistent with a shift in peripheral thyroid hormone metabolism away from active T3 production [220,221]. These responses are intensity-dependent, with higher exercise intensity associated with reductions in T3 despite elevations in T4 and TSH, suggesting diversion of T4 metabolism away from active T3 production [221]. In contrast, chronic exercise training may produce more sustained adaptations in thyroid hormone regulation. In a systematic review and meta-analysis of seven randomized controlled trials including adults with hypothyroidism, exercise interventions lasting more than 8 weeks were associated with reduced TSH and increased T4 levels when used alongside conventional treatment [222]. These findings suggest that acute exercise can transiently alter thyroid hormone availability in response to energetic stress, whereas longer-term training may improve thyroid hormone profiles in hypothyroid populations, although direct evidence in MASLD remains limited. In this context, thyroid–liver crosstalk may represent another component of exercise-induced systems-level mitochondrial remodeling, linking changes in hormone availability and tissue-specific thyroid hormone activation to hepatic lipid oxidation, bioenergetic flexibility, and mitochondrial quality control.

One mechanism by which exercise may alter local thyroid hormone action is tissue-specific regulation of deiodinase activity. In rodents during exercise, skeletal muscle D2 is rapidly upregulated via β-adrenergic signaling, increasing intracellular T3 production independent of circulating hormone levels [223]. This locally generated T3 activates nuclear TRs and supports exercise-induced mitochondrial adaptation. In skeletal muscle-specific D2 knockout rodent models, exercise fails to fully induce PGC-1α expression and mitochondrial enzyme activity, establishing a causal role for local T3 production in mitochondrial biogenesis [223]. In the liver, acute exercise may transiently suppress D1 activity and reduce systemic T3 availability [220], whereas chronic aerobic training increases hepatic D1 expression and circulating thyroid hormone levels in models of MASH [224]. In an atherosclerotic diet-induced MASH mouse model, aerobic exercise reduced steatosis, lobular inflammation, and fibrosis while increasing hepatic D1 expression and a T3-response gene signature, suggesting restoration of hepatic thyroid hormone signaling [224].

At the molecular level, thyroid hormone signaling works with exercise-responsive pathways to regulate mitochondrial function and metabolic adaptation. T3 binding to TRβ induces expression of PGC-1α, a regulator of mitochondrial biogenesis and fatty acid oxidation [204]. Exercise activates AMPK and SIRT1, which further enhance PGC-1α activity and downstream transcriptional programs [225]. Rather than functioning as separate pathways, thyroid hormone and exercise signaling converge on shared mitochondrial programs that regulate oxidative metabolism, substrate utilization, and mitochondrial quality control.

Within the liver, this convergence is particularly relevant to MASLD, where intrahepatic thyroid hormone dysregulation contributes to lipid accumulation and mitochondrial dysfunction. Mechanistically, thyroid hormone signaling promotes hepatic lipid catabolism through autophagy-mediated delivery of fatty acids to mitochondria for β-oxidation [204]. In a dietary mouse model of MASH, thyroid hormone treatment restored autophagy and mitochondrial biogenesis, increased fatty acid β-oxidation, and reduced hepatic triglyceride accumulation, oxidative stress, inflammation, and fibrosis [226]. Exercise further improves hepatic lipid handling in humans by reducing intrahepatic triglyceride content and improving hepatic insulin sensitivity, although evidence for effects on FFA flux and VLDL clearance is more variable and should be interpreted in the context of study design [227,228].

Thyroid hormone signaling may also intersect with exercise-responsive mitochondrial quality-control pathways. Both exercise and T3 activate AMPK-dependent autophagy signaling, linking mitochondrial turnover to energy sensing and metabolic stress [229,230]. Together with the MASH model evidence that T3 restores autophagy, lipophagy, mitochondrial biogenesis, and β-oxidation [226], these findings suggest that thyroid hormone signaling may support exercise-relevant pathways involved in mitochondrial turnover and oxidative stress resistance. However, whether exercise-induced changes in thyroid hormone signaling directly mediate hepatic mitochondrial quality control in human MASLD remains unresolved.

**Figure 4:**
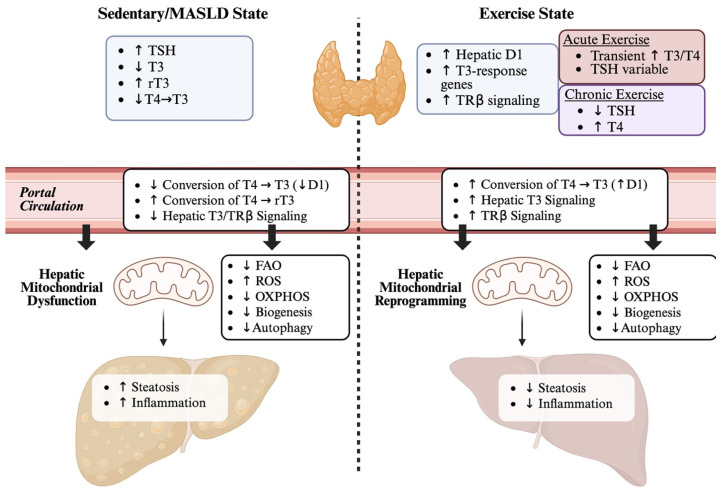
Exercise-Induced Mitochondrial Reprogramming of Thyroid–Liver Axis in MASLD. D1: type 1 deiodinase; FAO: fatty acid oxidation; OXPHOS: oxidative phosphorylation; ROS: reactive oxygen species; rT3: reverse triiodothyronine; TRβ: thyroid hormone receptor beta; TSH: thyroid-stimulating hormone. Created in BioRender.com. Jonas McCaffrey (2026). https://BioRender.com/ifgnbfp (accessed on 4 June 2026).

The distinctive feature of the thyroid–liver axis is local hormone activation. Exercise may influence MASLD by altering hepatic deiodinase activity and local T3/TRβ signaling, which converges with AMPK, SIRT1, PGC-1α, and autophagy pathways to regulate hepatic FAO and mitochondrial quality control. These adaptations may reduce hepatic lipid burden and limit progression toward inflammation and fibrosis, although direct evidence that exercise reverses intrahepatic hypothyroidism in human MASLD remains limited. A summary of exercise-induced mitochondrial reprogramming of the thyroid-liver axis in MASLD is depicted in Figure 4.

### 6.4. Therapeutic Implications of Thyroid Hormone Modulation in Exercise-Induced Metabolic Reprogramming

Therapeutic modulation of thyroid hormone signaling provides a clinically relevant framework for understanding how exercise-induced metabolic remodeling may be amplified in MASLD/MASH. Selective TRβ agonists, including resmetirom, have demonstrated reductions in hepatic fat content, improvements in liver histology, and favorable effects on lipid metabolism in patients with MASH [219]. These agents appear to improve hepatic lipid disposal while minimizing extrahepatic adverse effects associated with systemic thyroid hormone excess. The success of these therapies supports the concept that restoring hepatic thyroid hormone signaling may reverse key metabolic abnormalities in MASLD/MASH.

Exercise may augment these effects through several complementary mechanisms. First, exercise enhances AMPK and PGC-1α signaling, pathways that intersect closely with thyroid hormone-mediated mitochondrial biogenesis and oxidative metabolism [204,223]. Exercise also improves skeletal muscle insulin sensitivity and substrate utilization, thereby reducing hepatic lipid and glucose overflow [231]. In addition, exercise-induced myokines such as irisin and FGF21 may further enhance mitochondrial adaptation and lipid oxidation within hepatocytes. As highlighted in the previous section, exercise may influence thyroid hormone signaling directly through changes in deiodinase activity, peripheral thyroid hormone conversion, and tissue-level sensitivity to thyroid hormone signaling [223]. These effects appear dependent on exercise intensity, duration, nutritional state, and training status. Acute exercise can transiently increase circulating thyroid hormone concentrations and metabolic demand, while chronic training may improve tissue responsiveness and mitochondrial efficiency. These observations raise the possibility that exercise not only complements thyroid hormone signaling downstream but may also modulate upstream regulatory pathways that influence thyroid hormone action at the tissue level.

Importantly, exercise and THR-β agonists may exert complementary rather than redundant effects. Whereas pharmacologic activation primarily targets hepatic thyroid hormone signaling, exercise simultaneously remodels skeletal muscle metabolism, gut microbial function, systemic insulin sensitivity, and endocrine communication while also directly enhancing hepatic mitochondrial quality control. This raises the intriguing possibility that future therapeutic strategies combining structured exercise with thyroid hormone-based therapies could produce additive or synergistic improvements in mitochondrial function and metabolic health. Although direct clinical evidence remains limited, this represents an important area for future translational investigation.

An additional area of translational interest involves the use of low-dose thyroid hormone modulation as an adjunct to exercise-based interventions. Emerging clinical investigations evaluating low-dose levothyroxine in MASLD/MASH are based on the concept that modest enhancement of thyroid hormone signaling may improve mitochondrial fatty acid oxidation and reduce hepatic lipid accumulation without inducing systemic thyrotoxic effects [232]. Ongoing research in our laboratory has demonstrated that low-dose T3 is effective in increasing hepatic mitochondrial FAO and reversing MASLD in mice [233]. Based on these results, our group recently initiated a randomized double-blinded placebo-controlled clinical trial to test whether low-dose thyroxine (T4) is effective in improving the histological features in Veterans with biopsy-proven MASH (National Library of Medicine NCT05526144) [234]. In this context, exercise may enhance therapeutic responsiveness by simultaneously improving mitochondrial quality control, substrate handling, and oxidative capacity, while thyroid hormone modulation may amplify exercise-induced metabolic adaptation by increasing mitochondrial turnover and energy expenditure. Together, these observations support a conceptual model in which exercise and thyroid hormone signaling function as convergent regulators of mitochondrial remodeling and metabolic homeostasis. Future mechanistic studies and clinical trials will be needed to determine whether combined interventions produce additive or synergistic effects on hepatic steatosis, inflammation, fibrosis progression, and mitochondrial function, and whether biomarkers of mitochondrial adaptation and thyroid hormone responsiveness can support more individualized treatment approaches.

Collectively, thyroid hormone signaling should be viewed as an integral component of the systems-level network regulating hepatic mitochondrial reprogramming rather than as an isolated endocrine pathway. Through coordinated regulation of mitochondrial energetics, substrate oxidation, mitochondrial quality control, and metabolic flexibility, thyroid hormones interact with exercise-induced signaling originating from skeletal muscle, the gastrointestinal tract, and the liver itself to restore hepatocellular metabolic homeostasis. Recognition of these complementary mechanisms provides a conceptual framework for integrating lifestyle intervention with emerging thyroid-directed therapies in the management of MASLD/MASH.

## 7. Integrative and Translational Perspectives: Precision Exercise and Combination Therapy in MASLD/MASH

The mechanisms reviewed above support a model in which exercise acts as a systems-level mitochondrial remodeling stimulus rather than a nonspecific lifestyle intervention. Translating this framework into MASLD/MASH care will require combination strategies and precision biomarkers that capture inter-organ changes in substrate handling, lipotoxicity, inflammatory signaling, and mitochondrial quality control.

### 7.1. Combination Therapeutic Strategies Targeting Metabolic and Mitochondrial Remodeling

The emerging therapeutic landscape for MASLD/MASH supports combination strategies that target complementary layers of disease biology. Within the framework of this review, exercise should be viewed as a foundational systems-level intervention that improves hepatic disease not only through energy expenditure or weight loss, but through coordinated effects on skeletal muscle substrate handling, inter-organ signaling, hepatic lipid flux, inflammatory tone, and mitochondrial adaptation. Pharmacologic therapies may complement these effects by reducing systemic substrate burden or directly enhancing hepatic lipid disposal. Thus, the rationale for combination therapy is not simply additive weight loss, but the possibility of targeting multiple upstream drivers of mitochondrial stress that converge on hepatic steatosis, inflammation, and fibrotic progression.

GLP-1-based and dual incretin therapies primarily improve the systemic metabolic environment by reducing adiposity, improving glycemic control, increasing insulin sensitivity, and decreasing excess lipid and glucose delivery to the liver. In the SYNERGY-NASH trial, tirzepatide produced significantly higher rates of MASH resolution without worsening fibrosis compared with placebo, alongside weight loss and improvements in insulin sensitivity, lipid profiles, and glycemic control [235]. These effects may complement exercise, which increases cardiorespiratory fitness, skeletal muscle glucose uptake, fatty acid oxidation, and peripheral insulin sensitivity. In a randomized trial of patients with biopsy-proven MASH, energy restriction combined with supervised HIIT improved VO_2_ peak, peripheral insulin sensitivity, and liver disease resolution, supporting the concept that exercise redistributes excess nutrients toward skeletal muscle oxidation and away from hepatic lipotoxic storage [231]. Together, these findings suggest that incretin-based therapy and structured exercise may act on related but nonidentical components of MASLD/MASH pathogenesis as pharmacotherapy reduces systemic metabolic load, whereas exercise improves the oxidative and mitochondrial capacity needed to dispose of that load.

In contrast, THR-β agonism provides a more liver-directed strategy for improving hepatic lipid and mitochondrial metabolism. In the MAESTRO-NASH trial, resmetirom increased rates of MASH resolution and fibrosis improvement while reducing LDL cholesterol, triglycerides, and lipoproteins, with relatively neutral effects on body weight and insulin resistance [219]. This suggests that THR-β agonism targets hepatic lipid handling and mitochondrial energetics in a manner not redundant with either exercise or incretin-based therapy. A secondary analysis of MAESTRO-NASH further supports this complementary framework, showing that resmetirom efficacy was maintained in patients receiving background GLP-1 receptor agonist or SGLT2 inhibitor therapy, while weight loss of at least 5% was associated with greater improvements in MASH resolution, fibrosis, MRI-PDFF, and liver stiffness [236]. In this context, MRI-PDFF and liver stiffness provide practical clinical readouts of reduced hepatic lipid burden and tissue remodeling rather than direct measures of mitochondrial function. Their value in future combination trials will depend on pairing these endpoints with mechanistic biomarkers that clarify whether clinical improvement reflects enhanced substrate disposal, reduced lipotoxicity, improved thyroid hormone signaling, or mitochondrial remodeling.

Together, these studies support a translational model in which exercise, GLP-1-based therapies, and THR-β agonists act on overlapping but distinct layers of MASLD biology. Exercise functions as a systems-level mitochondrial remodeling stimulus by improving skeletal muscle substrate utilization, cardiorespiratory fitness, inter-organ endocrine signaling, and hepatic mitochondrial stress tolerance. Incretin-based therapies reduce adiposity and systemic substrate burden, thereby lowering the metabolic pressure placed on hepatic mitochondria, whereas THR-β agonism more directly promotes hepatic lipid disposal and mitochondrial energetics through liver-targeted thyroid hormone signaling. Future prospective trials should determine whether structured exercise combined with incretin-based therapy and/or THR-β agonism produces additive or synergistic effects on hepatic fat, MASH resolution, fibrosis, and patient-specific metabolic remodeling. Importantly, these trials should not only ask whether liver fat or histology improves, but whether combination therapy more effectively restores the mitochondrial and inter-organ networks that drive MASLD/MASH progression.

### 7.2. Precision Medicine, Multi-Omics, and Future Translational Trials

Future MASLD/MASH management will likely require precision approaches that match interventions to dominant disease mechanisms and patient-specific biology. A personalized framework for MASH care has emphasized the need to move beyond uniform treatment strategies by integrating lifestyle intervention, incretin-based therapies, liver-directed agents, and molecular profiling into mechanism-aligned care [237,238]. This approach is particularly relevant to exercise, which should not be viewed simply as a general lifestyle recommendation, but as a measurable systems-level mitochondrial intervention. Within this framework, exercise responsiveness may depend on whether a patient’s biology is driven by peripheral insulin resistance, skeletal muscle dysfunction, adiposity, gut-derived inflammation, altered thyroid hormone signaling, or impaired hepatic mitochondrial adaptation. Clinical trials could therefore assess whether exercise-induced changes in cardiorespiratory fitness, peripheral insulin sensitivity, substrate flux, and hepatic fat are accompanied by molecular changes in lipid metabolism, inflammation, mitochondrial function, and mitochondrial quality-control pathways.

Multi-omics platforms may help identify which patients are most likely to benefit from exercise alone or in combination with GLP-1-based therapy and THR-β agonism. Integrated transcriptomic and clinical-outcome datasets have shown that MASLD progression can be linked to disease-stage-specific gene expression signatures and prognostic transcriptional networks, including THR-β regulon activity as a suppressor of disease progression [239]. These findings suggest that pathway-level profiling could help identify patients with impaired hepatic thyroid hormone signaling who may respond preferentially to THR-β agonism, while other phenotypes may be more dependent on peripheral insulin resistance, adiposity, gut-derived inflammation, or impaired exercise responsiveness. Similarly, microbiome profiling may support precision exercise prescription. In a randomized trial of patients with MASLD and prediabetes, baseline gut microbial network structure predicted liver fat response to exercise intervention, supporting the concept that microbiome signatures may identify exercise-responsive and low-response subgroups [184]. Together, these studies support a precision framework in which molecular and microbial profiles are used not only to classify disease severity, but to determine which inter-organ pathways are most likely to limit mitochondrial remodeling in response to therapy.

Future translational trials should integrate clinical endpoints with mechanistic biomarkers that capture the inter-organ mitochondrial remodeling effects of exercise. Multi-matrix metabolomics in patients with MASLD showed that HIIT induced tissue-specific metabolic changes across adipose tissue, plasma, urine, and stool, including changes in amino acids, lipids, and glyco-conjugated bile acids, while improving VO_2_max, fasting glucose, and waist circumference without dietary change or weight loss [193]. These findings support the use of metabolomics, lipidomics, microbiome profiling, transcriptomic signatures, imaging endpoints such as MRI-PDFF and liver stiffness, and functional measures such as VO_2_peak to define biologic treatment response. However, these endpoints should be paired with mechanistic biomarkers to determine whether clinical improvement reflects enhanced skeletal muscle substrate handling, reduced gut-derived inflammatory stress, improved hepatic lipid oxidation, restored thyroid hormone signaling, or coordinated mitochondrial quality control. Such trials may help determine which patients require exercise alone, pharmacotherapy alone, or combination approaches targeting complementary components of metabolic and mitochondrial dysfunction. More broadly, this precision framework would allow future studies to test whether improvement in MASLD/MASH reflects true restoration of the inter-organ mitochondrial network that exercise is proposed to remodel.

## 8. Exercise-Induced Hepatic Mitochondrial Reprogramming: A Systems-Level Framework for MASLD/MASH

Figure 1 synthesizes the mechanistic evidence presented throughout this review into a systems-level framework of exercise-induced hepatic mitochondrial reprogramming. Although skeletal muscle, the gastrointestinal tract, thyroid hormone signaling, adipose tissue, and the liver have often been investigated independently, accumulating evidence suggests that these systems converge upon a common downstream target: hepatic mitochondrial function.

Within this framework, hepatic mitochondrial reprogramming represents the integrated remodeling of mitochondrial energetics, substrate utilization, quality-control pathways, redox homeostasis, and intracellular signaling that collectively restore hepatocellular metabolic resilience. Rather than representing a single signaling pathway, mitochondrial reprogramming should be viewed as an emergent biological property resulting from coordinated interactions among multiple endocrine, metabolic, inflammatory, and nutrient-sensing networks activated during exercise.

Exercise initiates this remodeling through repeated metabolic stress, leading to activation of conserved signaling pathways including AMPK, PGC-1α, sirtuins, thyroid hormone signaling, and mitochondrial quality-control mechanisms. These pathways collectively increase fatty acid oxidation, oxidative phosphorylation, ATP generation, mitochondrial biogenesis, and mitophagy while simultaneously reducing ROS accumulation, mitochondrial DNA damage, and inflammatory activation.

At the systemic level, skeletal muscle contributes through release of myokines and improved substrate utilization; the gut influences hepatic metabolism through microbial metabolites, bile acid signaling, intestinal barrier integrity, and immune regulation; thyroid hormones regulate mitochondrial energetics and metabolic flexibility; direct hepatic adaptations further reinforce improvements in mitochondrial efficiency. These diverse physiological responses converge upon common mitochondrial pathways, providing a mechanistic explanation for why exercise produces improvements in hepatic steatosis, inflammation, and fibrosis that frequently exceed those expected from weight loss alone.

Importantly, this conceptual framework also explains why exercise influences multiple stages of MASLD progression. Early in disease, enhanced mitochondrial oxidative capacity improves lipid handling and limits hepatic triglyceride accumulation. During established steatohepatitis, improved mitochondrial quality control, reduced oxidative stress, and attenuation of innate immune activation limit hepatocellular injury and inflammatory signaling. During more advanced disease, improved mitochondrial homeostasis may reduce stellate cell activation and slow fibrogenesis. Thus, hepatic mitochondrial reprogramming provides a unifying mechanism linking exercise to multiple pathological processes occurring throughout disease progression.

This framework also has important therapeutic implications. Rather than viewing exercise and pharmacologic therapy as competing approaches, they should be considered complementary interventions that target overlapping components of hepatic mitochondrial biology through distinct mechanisms. For example, thyroid hormone receptor-β agonists primarily enhance hepatic lipid oxidation and mitochondrial metabolism, whereas exercise simultaneously improves skeletal muscle metabolism, gut microbial function, systemic insulin sensitivity, endocrine signaling, and mitochondrial quality control. Such complementary mechanisms provide a strong biological rationale for future combination therapies integrating structured exercise with targeted pharmacologic interventions.

Finally, the concept of hepatic mitochondrial reprogramming shifts the therapeutic paradigm from treating isolated metabolic abnormalities toward restoring mitochondrial resilience as an integrated biological system. This systems-level perspective provides a mechanistic framework capable of integrating future discoveries in metabolism, immunology, endocrinology, and exercise physiology into a unified model of MASLD pathogenesis and treatment.

**Figure 5:**
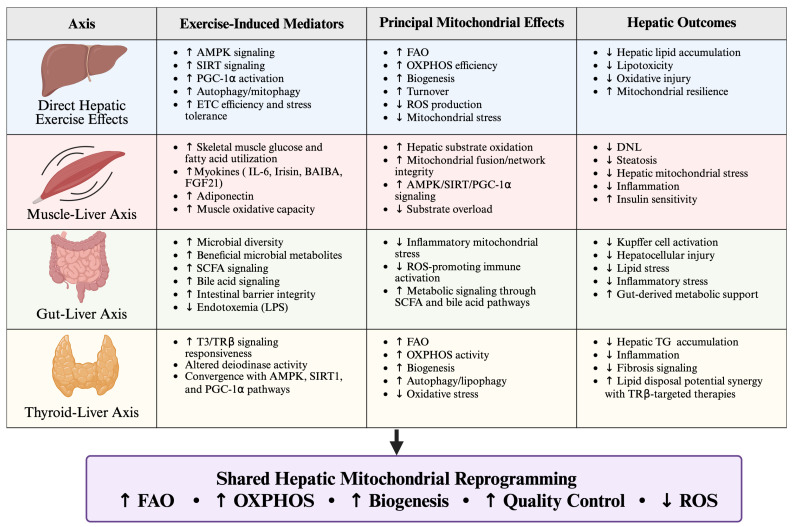
Convergent Pathways Linking Exercise-Induced Inter-Organ Signaling to Hepatic Mitochondrial Adaptation in MASLD/MASH. AMPK: AMP-Activated Protein Kinase; BAIBA: β-Aminoisobutyric Acid; DNL: De Novo Lipogenesis; ETC: Electron Transport Chain; FAO: Fatty Acid Oxidation; FGF21: Fibroblast Growth Factor 21; IL-6: Interleukin-6; LPS: Lipopolysaccharide; OXPHOS: Oxidative Phosphorylation; PGC-1α: Peroxisome Proliferator-Activated Receptor Gamma Coactivator-1 Alpha; ROS: Reactive Oxygen Species; SCFA: Short-Chain Fatty Acid; SIRT: Sirtuin; SIRT1: Sirtuin 1; T3: Triiodothyronine; TG: Triglyceride; TRβ: Thyroid Hormone Receptor Beta; ↑: Increase; ↓: Decrease. Created in BioRender.com. Jonas McCaffrey (2026). https://BioRender.com/1p3ik1m (accessed on 4 June 2026).

Taken together, the available evidence supports a paradigm in which hepatic mitochondrial reprogramming serves as the central mechanistic link between exercise and improvement in MASLD/MASH. Rather than viewing the muscle–liver, gut–liver, and thyroid–liver axes as independent pathways, they may be better understood as interconnected systems that converge upon mitochondrial adaptation. This integrated model provides a biologically plausible explanation for the broad therapeutic benefits of exercise and offers a framework for future mechanistic and translational investigations. A summary of this convergence framework, highlighting how exercise-responsive inter-organ pathways collectively promote hepatic mitochondrial reprogramming in MASLD/MASH is depicted in Figure 5.

## 9. Future Perspectives and Knowledge Gaps

Despite considerable advances in understanding exercise-mediated hepatic adaptation, numerous questions remain regarding the mechanisms through which exercise remodels mitochondrial biology in MASLD/MASH. Addressing these knowledge gaps will be essential for translating mechanistic discoveries into individualized therapeutic strategies.

First, greater emphasis should be placed on human mechanistic studies. Much of the current understanding of mitochondrial remodeling derives from animal models or cultured hepatocytes, whereas direct investigation of human liver tissue before and after structured exercise interventions remains limited. Advances in high-resolution respirometry, quantitative proteomics, metabolomics, lipidomics, and single-cell transcriptomics now provide opportunities to characterize exercise-induced mitochondrial remodeling in unprecedented detail. Future studies integrating these approaches with paired liver biopsies may identify molecular signatures predictive of therapeutic response.

Second, important heterogeneity likely exists among patients with MASLD/MASH. Factors including age, biological sex, ethnicity, obesity, sarcopenia, diabetes, genetic susceptibility, and baseline cardiorespiratory fitness may influence mitochondrial adaptation to exercise. Development of precision exercise prescriptions tailored to individual metabolic phenotypes represents an important future objective. Identification of circulating mitochondrial biomarkers capable of monitoring treatment response may further facilitate personalized intervention strategies.

Third, increasing attention should be directed toward interactions between exercise and emerging pharmacologic therapies. Recent approval of selective thyroid hormone receptor-β agonists, together with continued development of FGF21 analogues, GLP-1 receptor agonists, dual incretin therapies, and other metabolic agents, creates opportunities to investigate combination strategies targeting complementary components of hepatic mitochondrial biology. Whether structured exercise enhances pharmacologic responsiveness or permits lower therapeutic doses remains largely unexplored.

Fourth, the molecular pathways underlying hepatic mitochondrial reprogramming continue to evolve. Greater understanding is needed regarding mitochondrial dynamics, respiratory supercomplex remodeling, mitophagy, ferroptosis, mitochondrial DNA signaling, organelle communication, and interactions between mitochondria and the endoplasmic reticulum. These mechanisms may identify novel therapeutic targets while further refining our understanding of exercise-induced metabolic adaptation.

Finally, integration of artificial intelligence, systems biology, and computational network analysis offers new opportunities to identify regulatory networks governing hepatic mitochondrial adaptation. Such approaches may facilitate development of predictive models capable of integrating clinical, imaging, molecular, and physiological data to optimize individualized exercise prescriptions and improve long-term outcomes.

Collectively, these research priorities support a transition from empirical exercise recommendations toward mechanism-based precision medicine centered on restoration of hepatic mitochondrial health.

## 10. Conclusions

Exercise exerts profound therapeutic effects in MASLD/MASH that extend well beyond caloric expenditure or weight reduction. Accumulating evidence supports a broader paradigm in which exercise functions as a systems-level regulator of hepatic mitochondrial biology through coordinated interactions among skeletal muscle, the gastrointestinal tract, thyroid hormone signaling, and direct hepatic metabolic adaptation. These convergent pathways remodel mitochondrial energetics, enhance fatty acid oxidation, improve mitochondrial quality control, restore metabolic flexibility, and attenuate oxidative stress and inflammatory signaling.

The concept of exercise-induced hepatic mitochondrial reprogramming provides a unifying mechanistic framework linking these diverse physiological adaptations into a coherent model explaining the beneficial effects of exercise throughout the spectrum of MASLD/MASH. Rather than targeting isolated metabolic abnormalities, exercise restores mitochondrial resilience through integrated regulation of mitochondrial structure, function, and interorgan communication.

As pharmacologic therapies targeting mitochondrial metabolism continue to emerge, future management of MASLD/MASH will likely involve combination strategies integrating structured exercise with targeted metabolic interventions. Continued investigation of mitochondrial biology, precision exercise prescriptions, and translational biomarkers will further refine this framework and may ultimately enable personalized therapeutic approaches centered on restoration of hepatic mitochondrial health.

## Data Availability

No new data were created or analyzed in this study. Data sharing is not applicable to this article.
